# More than an attachment module: covalent inhibitor warheads influence BTK dynamics and function

**DOI:** 10.64898/2026.05.07.723540

**Published:** 2026-05-08

**Authors:** Raji E. Joseph, Robert G. Britton, David Yin-wei Lin, Julien Roche, Jeffrey A. Purslow, D. Bruce Fulton, Poowadon Fukasem, M. Paul Gleeson, Martin J. S. Dyer, Thomas E. Wales, Amy H. Andreotti

**Affiliations:** a Roy J. Carver Department of Biochemistry, Biophysics and Molecular Biology, Iowa State University, Ames, IA 50011, USA.; b Division of Cancer Sciences, Cancer Research Centre, College of Life Sciences, University of Leicester, Leicester LE1 9HN, UK.; c Department of Biomedical Engineering, School of Engineering, King Mongkut’s Institute of Technology, Ladkrabang, Bangkok 10520, Thailand.; d The Ernest and Helen Scott Haematological Research Institute, Leicester Cancer Research Centre, College of Life Sciences, University of Leicester, Leicester LE1 9HN, UK.; e Department of Chemistry and Chemical Biology, Northeastern University, Boston, MA 02115, USA.

**Keywords:** BTK, Bruton’s Tyrosine Kinase, BTK inhibitors, covalent warhead, kinase dynamics, Ibrutinib, Acalabrutinib, Zanubrutinib, Tirabrutinib, kinase inhibitor, kinase allostery, conformational heterogeneity

## Abstract

Covalent inhibitors are rapidly becoming the standard of care for treatment of a range of disease states. Covalent inhibitors bind irreversibly to their target using a reactive electrophile (or ‘warhead’). Acrylamide and 2-butynamide are the most commonly used cysteine targeting electrophiles. These warheads are chosen for their efficient and selective modification of the protein and are presumed to be otherwise functionally inert. Using a panel of BTK covalent inhibitors (Tirabrutinib, Acalabrutinib, Ibrutinib and Zanubrutinib), we show that the 2-butynamide warhead on Tirabrutinib and Acalabrutinib, unlike the acrylamide warhead on Ibrutinib and Zanubrutinib, induces conformational heterogeneity in key regions required for BTK signaling. Tirabrutinib or Acalabrutinib bound BTK adopt multiple conformational states that are in dynamic exchange, show increased binding to the substrate PLCγ and are less effective at inhibiting PLCγ signaling when compared to Ibrutinib. Swapping only the warheads between Tirabrutinib and Ibrutinib leads to a corresponding switch in BTK dynamics and inhibitor efficacy. The unanticipated warhead-specific allosteric effects raise interesting possibilities regarding inhibitor-specific mechanisms of resistance.

## INTRODUCTION

There are at least fifty covalent inhibitors approved for clinical use that target a wide range of proteins including viral proteases, G proteins and kinases (1). Covalent inhibitors are developed by attaching an electrophile (or warhead) to optimized high affinity reversible binders obtained from high throughput screens (2, 3). Notably, the same warheads are routinely used to generate covalent inhibitors and so, many clinically approved covalent inhibitors share the same warhead chemistry (4, 5). Acrylamide and 2-butynamide are the most commonly used cysteine modifier warheads (4). These electrophiles are chosen for their low reactivity which decreases toxicity arising from off-target, non-specific modifications (5).

The multi-domain, non-receptor kinase Bruton’s tyrosine kinase (BTK) (6) has emerged as an attractive target for covalent inhibitors. BTK is expressed primarily in cells of lymphoid or myeloid origin such as B cells, mast cells and macrophages where it functions downstream of immune receptors including the B cell receptor (BCR) and Fc receptor (6–8). BTK inhibitors are used in the treatment of multiple B cell malignancies and are being evaluated for the treatment of myeloid disorders such as rheumatoid arthritis and multiple sclerosis (9–11). Seven of the eight approved drugs that target BTK (Ibrutinib, Acalabrutinib, Zanubrutinib, Tirabrutinib, Rilzabrutinib, Remibrutinib and Orelabrutinib) are covalent inhibitors (12). All of these inhibitors have demonstrated efficacy and a pipeline of additional therapeutically valuable BTK inhibitors are at various stages of clinical development (8, 13). Motivating this drug pipeline is the fact that acquired inhibitor-specific resistance mutations arise in clinical settings (14). As treatment options continue to develop it is important to understand how the chemical features of available inhibitors affect the target BTK protein and ensuing downstream responses.

**Figure 1:**
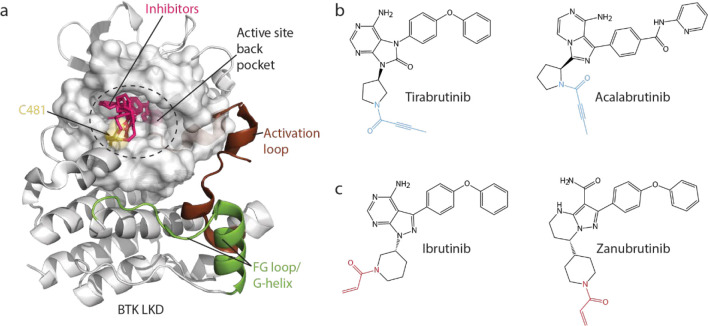
BTK inhibitors (a) Structure of Ibrutinib bound BTK (PDB ID: 5P9J) overlaid with the structures of Tirabrutinib, Acalabrutinib and Zanubrutinib (pink). The kinase active site is shown as a space filling model. Covalent inhibitors bind to BTK C481 (yellow) and extend into the active site back pocket. The kinase activation loop (brown) and the FG loop/G-helix (green) regions are labeled. (b,c) Structures of covalent BTK inhibitors used in this study. The electrophilic warheads are colored blue (2-butynamide) or red (acrylamide).

Crystal structures of BTK/inhibitor complexes show a common binding mode: all utilize BTK C481 for covalent attachment, with the binding moiety extending into the kinase active site ‘back’ pocket (Figure 1a). Covalent attachment of the BTK inhibitors Tirabrutinib and Acalabrutinib is mediated via the 2-butynamide warhead whereas Ibrutinib and Zanubrutinib have an acrylamide warhead (Figure 1b,c (4, 15)). While these distinct warheads offer differences in reactivity, once the covalent bond is formed it is presumed that the warheads on different BTK inhibitors serve solely as attachment modules. Whether the warheads on BTK inhibitors have additional impacts on BTK has not been evaluated.

Our previous solution studies using Nuclear Magnetic Resonance (NMR) spectroscopy and Hydrogen-Deuterium Exchange-Mass Spectrometry (HDX-MS) have shown that a subset of BTK inhibitors have unanticipated long-range effects on the conformation of full-length enzyme (16, 17). Inhibitor characteristics that cause these structural changes and the functional consequences of inhibitor induced structural changes remain unexplored. Here we probe the determinants of specific inhibitor-induced allosteric changes in the BTK kinase domain using a combination of HDX-MS, NMR and computational analysis. We find that unique dynamic features induced by some inhibitors and not others have functional consequences. The observed conformational preferences are determined solely by the chemical nature of the covalent warhead. Inhibitors that utilize the 2-butynamide warhead, unlike the acrylamide warhead, induce multiple kinase domain conformational states resulting in increased deuterium uptake within the kinase domain, enhanced binding to the BTK substrate Phospholipase Cγ (PLCγ), and less efficient inhibition of PLCγ signaling. Given the growing popularity of covalent inhibitors, the unanticipated allosteric effects driven by the nature of the covalent attachment should be considered in future inhibitor design and development.

## RESULTS

### Tirabrutinib and Acalabrutinib induce functionally important long-range conformational effects in the BTK kinase domain.

**Figure 2:**
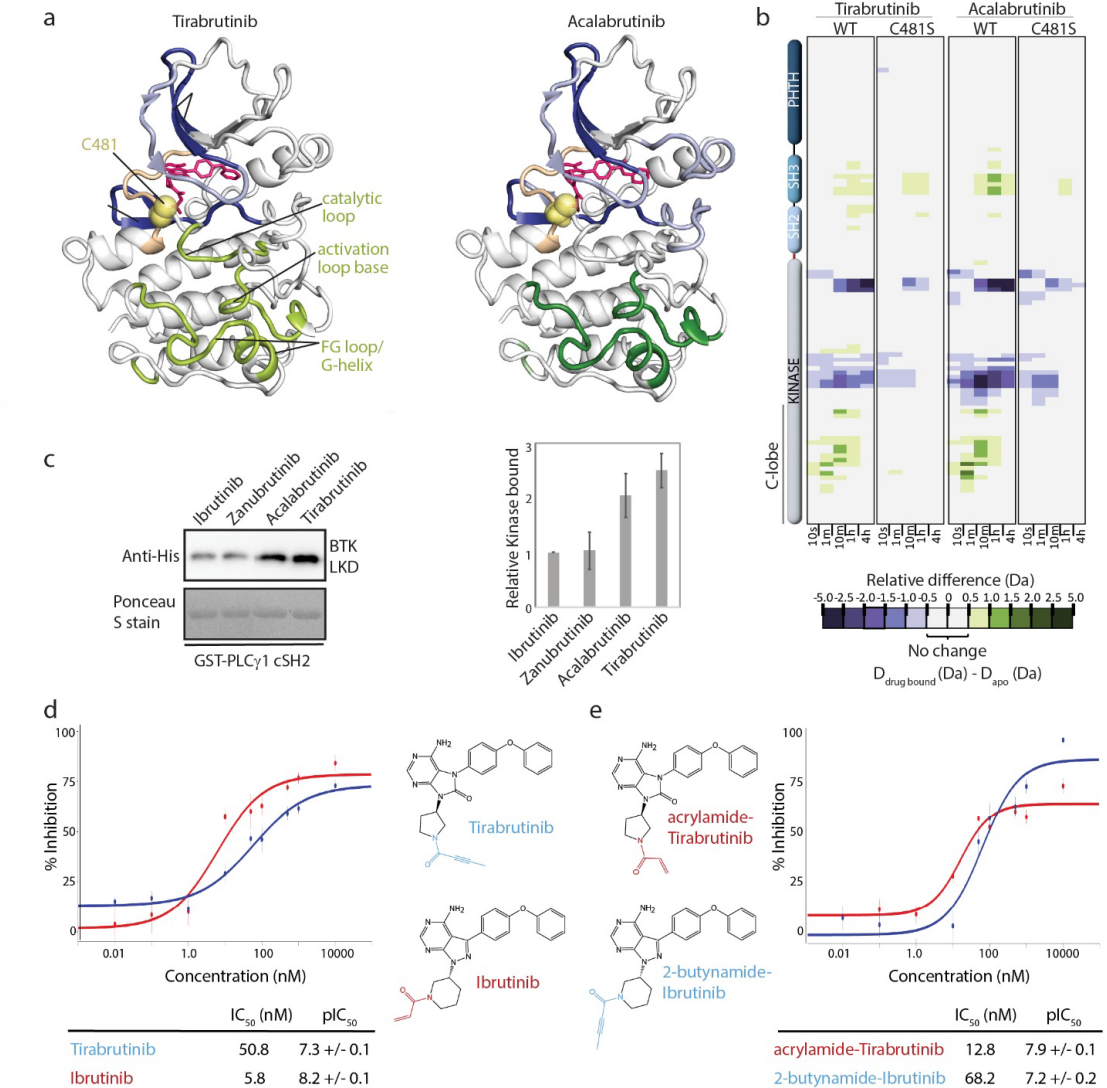
Dynamic allostery and functional effects of the distinct warheads. (a) Mapping the HDX-MS changes induced by inhibitor binding onto the structure of the BTK kinase domain. Differences > 1.0 Da are dark blue (decrease) or dark green (increase); differences 0.5 Da-1.0 Da are light blue (decrease) and light green (increase). Inhibitors are pink and C481 is yellow. Regions of increased deuterium uptake in the kinase domain C-lobe are labeled. (b) Covalent attachment of BTK with Tirabrutinib and Acalabrutinib is required to observe the dynamic changes in the C-lobe. Relative deuterium level of peptides in apo BTK is subtracted from the deuterium level of the corresponding peptide from each drug-bound form (D_drug-bound_-D_apo_); scale shows magnitude of differences. Peptic peptides are shown from BTK N- to C-terminus, top to bottom, and sample time in deuterium is shown left to right. (c) Tirabrutinib or Acalabrutinib bound BTK linker-kinase domain (LKD) shows increased binding to PLCγ as compared to Ibrutinib and Zanubrutinib bound BTK LKD. Anti-His detects bound BTK, Ponceau stain shows total PLCγ cSH2. Bands were quantified and plotted as a histogram with the error bars representing standard deviation. Data shown is the average of three independent experiments. (d) Ibrutinib (red) is more effective than Tirabrutinib (blue) at inhibiting calcium flux in BCR stimulated Ramos B cells. Structures of both inhibitors are shown highlighting the 2-butynamide (blue) and acrylamide (red) warheads. Half maximal inhibitory concentration (IC_50_) values and −log_10_ IC_50_ (pIC_50_) values ± SD (n=6) of calcium flux inhibition are listed. (e) Data are as described in (d); IC_50_ for acrylamide-Tirabrutinib (red) is lower than 2-butynamide-Ibrutinib (blue).

HDX-MS analysis of covalent BTK inhibitors Tirabrutinib, Acalabrutinib, Ibrutinib, and Zanubrutinib have shown that drug binding to full-length BTK induces an increase in deuterium uptake in the SH3 and SH2 domains and a decrease in the kinase N-lobe surrounding the active site (Figure 2a,b, Supp. Fig. S1a, (16)). HDX-MS data also reveal differences; binding of Tirabrutinib or Acalabrutinib to BTK (but not Ibrutinib or Zanubrutinib) results in an increase in deuterium uptake across the kinase domain C-lobe (Figure 2a,b Supp. Fig. S1a,b). The differences in the C-lobe among the panel of BTK/inhibitor complexes are not evident in crystal structures of the BTK/drug complexes (Supp. Fig. S2a-e).

The inhibitor specific dynamic differences in the FG loop/G-helix region of the C-lobe is of particular interest, as this region has been previously identified as a docking site required for BTK mediated phosphorylation of its substrate PLCγ (18, 19). To test whether the inhibitor induced conformational/dynamic changes in the FG loop/G-helix might affect substrate binding, we compared the interaction of PLCγ with the different covalent inhibitor/BTK complexes. His-tagged BTK linker-kinase domain (LKD) bound to Ibrutinib, Zanubrutinib, Acalabrutinib or Tirabrutinib was incubated with GST-tagged PLCγ1 cSH2 (minimal fragment required for direct PLCγ/BTK interaction). Tirabrutinib or Acalabrutinib bound BTK interact more effectively with PLCγ compared to Ibrutinib or Zanubrutinib bound BTK (Figure 2c). This finding suggests that the conformational/dynamic features unique to the FG loop/G-helix in the BTK/Tirabrutinib or BTK/Acalabrutinib complexes (Figure 2a,b) directly affect PLCγ substrate interaction.

The observed differences in PLCγ binding are interesting in the context of previous studies reporting lower efficacy (higher IC_50_) for Acalabrutinib compared to Ibrutinib in terminating downstream PLCγ signaling in B cells (20, 21). To first probe whether Tirabrutinib, which like Acalabrutinib contains the 2-butynamide warhead, is similarly less effective than Ibrutinib we measured IC_50_ values for both drugs. Following previous reports for Acalabrutinib, we find that Tirabrutinib is less effective (IC_50_=50.8 nM) at terminating calcium signaling post BCR stimulation compared to Ibrutinib (IC_50_=5.8 nM), raising the possibility that the 2-butynamide warhead containing BTK inhibitors might generally be less efficient at terminating PLCγ signaling (Figure 2d). The difference in IC_50_ values we measure for Tirabrutinib versus Ibrutinib (approaching ten-fold) is on the same order of magnitude reported previously for Acalabrutinib and Ibrutinib (22).

### The covalent attachment site is responsible for conformational dynamics in the kinase domain C-lobe.

There are several chemical differences between Tirabrutinib and Ibrutinib, pyrrolidine versus piperidine (attached to 2-butynamide or acrylamide, respectively) as well as differences between the aromatic core structures of each drug. To begin to probe the molecular determinants of the observed differences in kinase domain dynamics and cellular efficacies we tested the importance of the covalent bond between inhibitor and C481. Deuterium uptake changes in the wildtype BTK kinase domain C-lobe are abolished upon addition of either Tirabrutinib or Acalabrutinib to the BTK C481S mutant (Figure 2b). These data support the conclusion that the C481 mediated covalent bond is required to induce the allosteric effects observed in the C-lobe (FG loop/G-helix) of the BTK kinase domain prompting a closer examination of the different warheads.

### Swapping electrophilic warheads switches the dynamic allostery and functional profiles of BTK inhibitors.

Two drug analogs, a Tirabrutinib analog containing the Ibrutinib warhead (denoted acrylamide-Tirabrutinib) and an Ibrutinib analog containing the Tirabrutinib warhead (2-butynamide-Ibrutinib, (Figure 2e)), were synthesized to determine the extent to which the different attachment modules affect potency in the B cell assay. The efficacy of each analog in terminating calcium signaling post BCR stimulation was measured as for the parent Tirabrutinib and Ibrutinib compounds. The IC_50_ value for the 2-butynamide-Ibrutinib analog is similar to that of Tirabrutinib and the acrylamide-Tirabrutinib analog IC_50_ is similar to that of Ibrutinib (Figure 2e). Swapping only the warheads between Tirabrutinib and Ibrutinib switches the efficacy of each drug.

**Figure 3:**
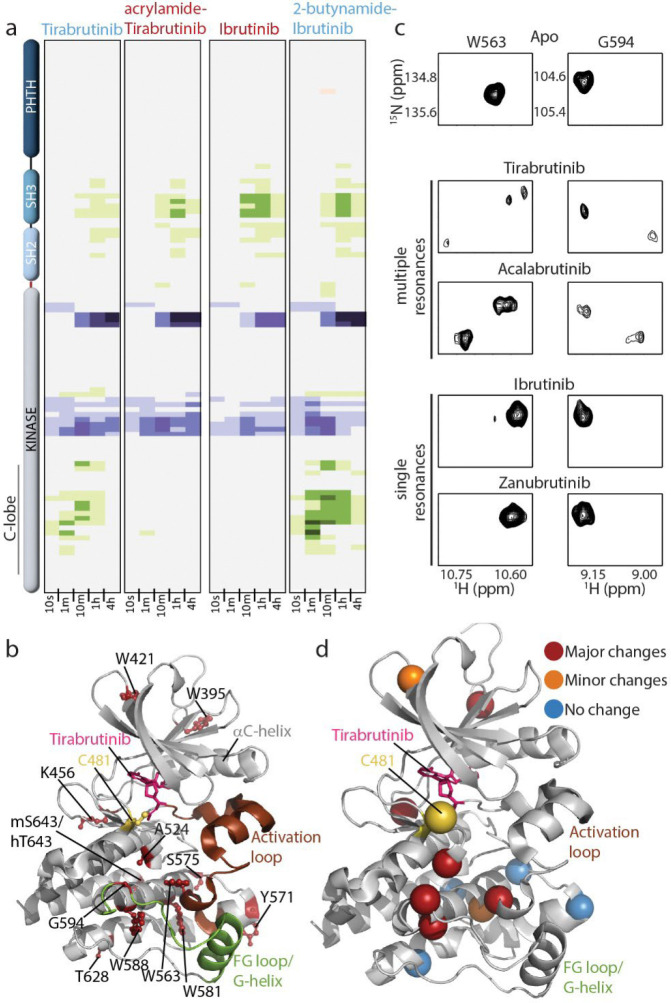
Swapping the electrophilic warhead between Tirabrutinib and Ibrutinib switches the solution behavior of the corresponding BTK/inhibitor complexes. (a) HDX-MS data for Tirabrutinib and Ibrutinib and corresponding analogs. (b) Residues with NMR assignments are shown as red sticks and labeled on the structure of Tirabrutinib bound BTK kinase domain. (c and d) Tirabrutinib or Acalabrutinib bound BTK adopt multiple conformations. (c) Select resonances from the ^1^H-^15^N TROSY-HSQC spectra of apo BTK kinase domain and four inhibitor complexes. (d) Kinase domain residues that give rise to multiple peaks upon binding of Tirabrutinib or Acalabrutinib are mapped onto the structure. Red spheres indicate well resolved multiple peaks observed for both drug/BTK complexes, orange are residues that show conformational heterogeneity in one or the other BTK/inhibitor, and blue represent residues that do not show multiple resonances.

We next reasoned that the observed switch in functional efficacy might correlate with the presence/absence of allosteric changes measured by HDX in the BTK kinase domain C-lobe (Figure 2b, Supp Fig. 1a,b). To test this idea, we compared the HDX profiles for Tirabrutinib, acrylamide-Tirabrutinib, Ibrutinib and 2-butynamide-Ibrutinib and find that only the 2-butynamide containing inhibitors cause increased deuterium uptake in the kinase domain C-lobe (Figure 3a). In contrast, all four inhibitors induce nearly identical HDX differences across the SH3-SH2 domain and surrounding the BTK active site (Figure 3a). As observed for the parent compounds, crystal structures of acrylamide-Tirabrutinib and 2-butynamide-Ibrutinib bound to the BTK kinase domain show no differences in the FG loop/G-helix region and covalent attachment is necessary; no changes in HDX are observed in the kinase domain C-lobe upon addition of acrylamide-Tirabrutinib or 2-butynamide-Ibrutinib to the BTK C481S mutant (Supp. Fig. S3a,b). Thus, the product of covalent attachment to the 2-butynamide warhead is necessary and sufficient to induce allosteric changes in the BTK kinase domain C-lobe.

To compliment the HDX-MS data, we applied NMR spectroscopic approaches to study the conformational effects of drug binding to the BTK kinase domain. Our previously reported backbone assignments for the apo BTK kinase domain (23) allowed unambiguous assignments for a subset of residues providing NMR probes distributed across the kinase domain (Figure 3b, Supp. Fig. S4). Clear differences in NMR spectral features emerge for the Tirabrutinib and Acalabrutinib complexes compared to the BTK kinase domain bound to Ibrutinib or Zanubrutinib. Binding of the 2-butynamide containing drugs (Tirabrutinib and Acalabrutinib) induces multiple NMR resonances for a subset of residues whereas the acrylamide containing inhibitors do not induce multiple resonances (Figure 3c, Supp. Fig. S5a). Conformational heterogeneity is abolished without the covalent bond between BTK and either Tirabrutinib or Acalabrutinib (Supp. Fig. S5b,c).

The appearance of multiple resonances for individual amino acids in the BTK kinase domain suggests slow conformational exchange and so we carried out ZZ-exchange experiments on BTK bound to Tirabrutinib. Off-diagonal peaks were detected providing direct evidence of exchange on the millisecond timescale (Supp. Fig. S5d). High-pressure NMR has also previously been used to characterize transient intermediate states (24). More broadly, hydrostatic pressure favors conformations with smaller partial molar volumes and can slow the rate of interconversion, thereby simplifying complex exchange processes (25, 26). We therefore applied high-pressure NMR to the Tirabrutinib–BTK complex to define the multi-conformational states revealed by peak splitting and find that increasing pressure causes all of the split resonances to merge into single peaks at 2000–2500 bar, consistent with a dynamic equilibrium between multiple conformational states at ambient pressure (Supp Fig. S5e). The spectral changes observed at high pressure are reversible when the pressure is returned to 1 bar. By contrast, residues that do not exhibit peak splitting upon inhibitor binding exhibit only gradual chemical shift changes with pressure (Supp. Fig. S5e), an effect attributed to non-specific preferential hydration of the backbone that produces small downfield shifts universally observed in proteins under pressure (27). These observations confirm that slow conformational exchange is localized to specific residues of the Tirabrutinib-bound kinase domain, while the remainder of the domain behaves as a single, well-defined conformation.

Residues showing the most pronounced peak splitting are localized to the kinase domain C-lobe; the same region of the kinase domain that exhibit increased deuterium uptake by HDX-MS (FG loop/G-helix and base of activation loop) (Figure 3d). BTK residues in other regions of the kinase domain exhibit less pronounced spectral differences, either the presence of multiple peaks in spectra of only one and not both Tirabrutinib or Acalabrutinib bound BTK samples or appear as single peaks (Figure 3d, Supp. Fig S5a). Distinct conformational states of the FG loop/G-helix region could cause the observed increase in deuterium uptake in Tirabrutinib or Acalabrutinib bound BTK and in turn may drive the differences in substrate engagement and efficacy in terminating B cell signaling.

### Computational analysis of inhibitor binding to BTK.

**Figure 4:**
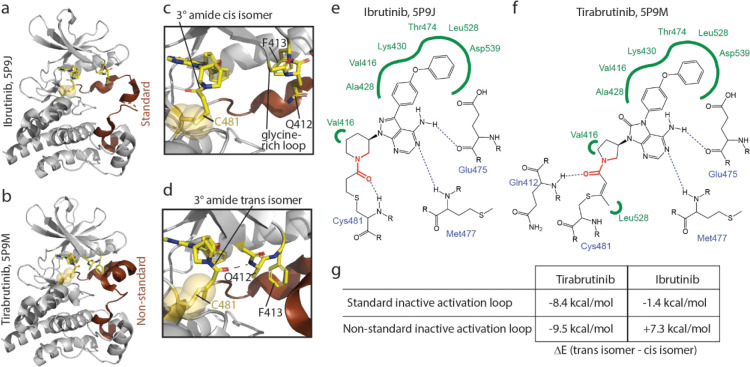
Density Functional Theory (DFT) calculations support the presence of multiple protein conformational states in Tirabrutinib bound BTK. (a and b) Structures showing the ‘standard’ and ‘non-standard’ inactive activation loop conformations in (a) Ibrutinib versus (b) Tirabrutinib bound BTK LKD. (c,d) Zoomed view of the BTK active site structure showing the cis amide bond isomer for Ibrutinib (c) and the trans amide bond isomer for Tirabrutinib (d). Also labeled are C481 and two residues (Q412 and F413) on the glycine rich loop. (e,f) 2D representations of (e) Ibrutinib and (f) Tirabrutinib binding poses adapted from Proteins*Plus*, PoseView and PoseEdit (44). The tertiary amide bond in each inhibitor is colored red. (g) Electronic energies obtained from DFT calculations are shown as ΔE values (kcal/mol) for Tirabrutinib and Ibrutinib in their cis versus trans amide bond configurations bound to the BTK kinase domain active site with either the ‘standard’ or ‘non-standard’ inactive activation loop conformations. For each protein conformation, ΔE is defined as the calculated electronic energy of the inhibitor docked with the trans amide bond minus the energy of the inhibitor docked with the cis amide bond. The more thermodynamically favorable a given structure is, the more negative the energy value.

Having confirmed that 2-butynamide containing inhibitors induce multiple exchanging conformational states in the BTK kinase domain, we set out to use computational approaches to ascertain the source of the observed conformational heterogeneity. Analysis of over 100 x-ray structures of BTK bound to covalent active site inhibitors deposited in the Protein Data Bank shows that all but one exhibit the standard conformation associated with the inactive kinase activation loop (A-loop) (Figure 4a). One structure, that of Tirabrutinib bound to the BTK kinase domain, reveals a unique A-loop conformation, which we refer to as the ‘nonstandard’ inactive A-loop conformation (Figure 4b). The resolution of these two structures (1.08Å and 1.41Å, respectively) allows for a detailed description of inhibitor orientations. The tertiary amide of Ibrutinib (in the context of the standard inactive A-loop) adopts the cis isomer (Figure 4c,e). In contrast, the trans amide bond isomer is observed for Tirabrutinib whether the A-loop adopts the standard or non-standard conformation (Figure 4d,f and see PDB ID: 8FF0). In the latter, the trans amide isomer in Tirabrutinib permits formation of a hydrogen bond between the amide carbonyl of Tirabrutinib and the backbone amide NH of Q412 on the glycine-rich loop of the kinase domain (Figure 4d,f). This interaction allows hydrophobic packing of the adjacent BTK F413 sidechain into a pocket that is formed by the non-standard activation loop conformation (Figure 4d). The cis amide bond isomer observed in structures of Ibrutinib bound to BTK show no contacts between drug and the glycine-rich loop (Figure 4c,e).

To further understand the interplay between inhibitor and protein conformational preferences, we generated quantum mechanics (QM)-based cluster models of the standard inactive A-loop and non-standard inactive A-loop consisting of ~450 atoms surrounding the ATP binding site (Supp. Fig. S6). Tirabrutinib and Ibrutinib were docked to both enzyme conformations, in both the cis and trans amide-bond conformations (i.e. 4 states per inhibitor). Geometry optimization using Density Functional Theory (DFT) methods were performed and the energetics associated with each of the four states were compared (Figure 4g). For both A-loop conformations, the Tirabrutinib tertiary amide bond shows an energetic preference for the trans isomer in the BTK active site (Figure 4g). In contrast, the calculations suggest that the tertiary amide bond within Ibrutinib shows a marginal energetic preference for the trans isomer in the context of the standard inactive conformation of the A-loop and a clear preference for cis in the non-standard inactive A-loop conformation. Overall, these findings are consistent with the observation that Tirabrutinib and not Ibrutinib has been crystalized in both the standard and non-standard activation loop states.

From the computational experiments and available crystal structures we can conclude that the tertiary amide bond of Tirabrutinib prefers to adopt the trans conformation within the ATP-binding site of BTK, irrespective of the A-loop conformation, while Ibrutinib can adopt both the cis and trans amide bond states. We postulate that the more limited conformational preference of the amide bond in Tirabrutinib may be the result of the adjacent alkene bond as opposed to the more flexible alkyl present in Ibrutinib/BTK (Figure 4e,f). Of course, like the tertiary amide bond, the alkene bond in the covalent adduct of Tirabrutinib (or Acalabrutinib) can also adopt Z and E isomers, albeit non-interconverting and determined by the reaction geometry. However, modeling the alkyne precursors of Tirabrutinib (or Acalabrutinib) into the BTK active site reveals only one orientation of the triple bond that allows for a productive nucleophilic attack by the C481 thiol. This is consistent with the observation that only the Z double bond isomer is captured in crystal structures of the covalent Tirabrutinib/Acalabrutinib BTK complexes and that the multiple conformations observed in solution are in dynamic exchange (Supp. Fig. S5d,e). Additionally, the methyl substituent on the resulting Z carbon-carbon double bond makes favorable hydrophobic contacts with the sidechain of L528 (Figure 4f); no such interactions are present in the Ibrutinib complex (Figure 4e). Collectively, we find that Tirabrutinib (and by extension Acalabrutinib) appear to adopt a single conformation in the active site of BTK (trans amide and Z alkene) and these 2-butynamide containing inhibitors stabilize at least two different BTK conformations (standard and non-standard A-loop) likely providing an explanation for the multiple NMR peaks observed for these complexes.

## DISCUSSION:

The reliance of B cell cancers on BCR signaling has made BTK inhibition an effective treatment approach for specific B cell malignancies (28, 29). The current collection of BTK inhibitors provide numerous treatment options resulting in complex clinical considerations in selection of specific treatment approaches. In addition, both Ibrutinib and Acalabrutinib are now approved for use in combination therapy with the BCL-2 inhibitor venetoclax with emerging discussions around which combination may be more effective (30–32). It will take time for long-term data to emerge on benefits/risks of one BTK inhibitor over another but it remains important to continue to advance our understanding of BTK inhibitor mechanism of action in the clinic, in B cells and at the molecular level.

The crystal structures of BTK in complex with the range of available covalent inhibitors are strikingly similar. It has therefore been assumed that the nature of the electrophile (and in turn the chemical structure of the covalent attachment to C481) does not affect the structure, dynamics or signaling capacity of inhibitor bound BTK. Our data demonstrate that covalent attachment of the 2-butynamide warhead on Tirabrutinib and Acalabrutinib to C481 (and not the acrylamide warhead) leads to allosteric changes in the dynamics and structure of the C-lobe of the BTK kinase domain. Tirabrutinib or Acalabrutinib bound BTK complexes both adopt multiple conformational states that are in slow exchange; this conformational heterogeneity correlates with an increase in binding to the BTK substrate PLCγ *in vitro*. The stronger association between drug bound BTK and PLCγ likely destabilizes the autoinhibited conformation of PLCγ and increases the accessibility of PLCγ for phosphorylation/activation by uninhibited BTK or other kinases. These differences are not sufficient to dramatically affect efficacy; all of these BTK inhibitors are effective in patients. It is however reasonable to imagine that the enhanced BTK/PLCγ interaction and downstream signaling could create distinct opportunities for emergent mutations (in PLCγ or other downstream molecules) that drive resistance mutations specific to the 2-buynamide warhead (33, 34). Additional real-world data tracking resistance mutation profiles that occur under distinct drug pressures are needed to test this hypothesis.

The idea that differences in BTK inhibitor binding may drive differences in downstream signaling despite potent inhibition of BTK catalytic activity is borne out in the literature. In one study, Bender *et. al*., report that inhibitor binding mode (and the resulting conformation/phosphorylation status of the BTK kinase) results in differential compound potency against FcεR versus BCR signaling (35). A more recent study also focused on comparing inhibitors that occupy different regions of the active site (back pocket, hinge region and H3 pocket). In this work, Li *et al.* (22) examined the performance of different inhibitors in the context of acquired resistance. Specifically, in an induced model of resistance, these authors found that Ibrutinib retained cytotoxic activity in the nanomolar range while Acalabrutinib (as well as the front pocket and hinge binders) did not (22). Li and co-workers also demonstrated that different BTK inhibitors trigger distinct downstream functional effects via non-catalytic scaffolding interactions. That work was predicated on our earlier biophysical studies showing that BTK inhibitors that bind the back pocket, hinge region or H3 (front) pocket cause distinct allosteric structural and dynamic effects in the remote non-catalytic regions of the BTK (16, 17). Our results now show that even the closely related back pocket binders (Ibrutinib/Zanubrutinib versus Acalabrutinib/Tirabrutinib) trigger different conformational preferences in BTK due solely to differences in warhead structure.

Use of the acrylamide and 2-butynamide warheads is not limited to development of BTK inhibitors. Currently, at least eleven covalent inhibitors that use either the acrylamide or 2-butynamide warheads are approved for clinical use (4, 36). These covalent inhibitors target a variety of proteins including G proteins such as KRAS and kinases such as epidermal growth factor receptor (EGFR), fibroblast growth factor receptor (FGFR) and Janus (JAK) kinases in addition to BTK (36). The success of these warheads has reinvigorated the search and development of other electrophiles for the design of covalent inhibitors. The focus of these development efforts has primarily been on optimizing reactivity and selectivity, as well as expanding chemoselectivity (37). Our findings suggest that different electrophilic warheads also impart distinct conformational/dynamic effects on their protein targets that can influence signaling outcomes.

## MATERIALS AND METHODS:

### Constructs and reagents:

All bacterial expression BTK and PLCγ1 cSH2 constructs have been described previously (33, 38, 39). Acalabrutinib, Zanubrutinib and Tirabrutinib were purchased from MedChem Express. Ibrutinib was purchased from Selleckchem. The acrylamide-Tirabrutinib and 2-butynamide-Ibrutinib analogs were synthesized as described in Supp. Data Files 1 and 2.

### Protein expression, purification and in vitro assays:

Expression and purification of the PLCγ1 cSH2 and BTK kinase domain have been described previously (33, 38, 39). BTK/PLCγ interactions assay: 0.1 μM His-tagged BTK LKD was preincubated with 1.0 μM BTK inhibitor (Ibrutinib, Zanubrutinib, Acalabrutinib or Tirabrutinib) for 30 min at RT in pull-down buffer (20 mM Tris-HCl (pH 7.3), 150 mM Sodium chloride, 10% glycerol and 2% DMSO). 0.5 μM GST-tagged PLCγ1-cSH2 domain and 10 μL glutathione beads were added to the inhibitor bound BTK LKD and mixed for 2 hours at 4°C. The beads were washed thrice with the pull-down buffer and boiled in SDS-PAGE loading dye. The samples were separated by SDS-PAGE and western blotted with an Anti-His antibody (Millipore Sigma) (38). Western blots were developed by chemiluminescence and images captured using the ChemiDoc^™^ gel imaging system (Biorad). The intensity of the bands was quantified using Image Lab (Biorad). Data from three independent experiments were averaged. The intensity of Ibrutinib bound BTK LKD was set to 1 and the intensity of the other inhibitor bound BTK LKD is reported relative to Ibrutinib bound BTK.

### Calcium flux assay and IC_50_ determination:

Ramos B cells (ATCC, # CRL-1596) were maintained in RPMI 1640 medium (Gibco, #A1049101) supplemented with 10% FBS (Thermo Fisher Scientific) and Penicillin/Streptomycin (Thermo Fisher Scientific) at 37°C/5% CO_2_. One day prior to the calcium assay, the cells were rinsed once with PBS and plated in RPMI 1640 medium containing 1% FBS in 96 well clear bottom black plates (Corning, CellBIND) at a density of 100,000 cells/well and incubated for 18 h at 37°C/5% CO_2_. Inhibitors (0.1% DMSO final concentration) and the FLIPR calcium 6 assay reagent (Molecular Devices) were added to the cells for one hour at 37°C/5% CO_2_. Cells were stimulated with 5 μg/ml of F(ab’)_2_ anti-human IgM (Southern Biotech, # 2022–01) and fluorescence was monitored at 37°C for 6 minutes (Ex. 480 nm, Em. 545 nm) on a Tecan Spark plate reader. IC_50_ values for calcium flux inhibition were calculated using the AAT Bioquest IC_50_ calculator (40).

### NMR spectroscopy:

Uniformly ^15^N labeled BTK samples were produced as described previously (38). All NMR spectra were acquired at 298 K on a Bruker AVIII HD 800 MHz spectrometer equipped with a 5 mm TCI (H/F)CN z-shielded gradient triple resonance cryoprobe, operating at a ^1^H frequency of 800.37 MHz. NMR samples with inhibitors consisted of 150 μM ^15^N labeled BTK, mixed with 200 μM inhibitor and 2% DMSO unless indicated otherwise. All data were analyzed using NMRViewJ (41). Methods for high pressure NMR and ZZ-exchange experiments (and x-ray crystallography) are provided in Supplemental Data File 1.

### HDX-MS:

Procedures for HDX-MS of BTK have been described previously (16, 17). Experimental parameters are provided in the Supp. Data File 3 (42). All HDX-MS data have been deposited to the ProteomeXchange Consortium via the PRIDE (43) partner repository with the dataset identifier PXD069107. Comparisons have been made to previously published HDX MS data (taken from reference (16), identified with an asterisk in Supp. Data File 3 and a dataset identifier PXD047865 in the PRIDE partner repository). Deuterium incorporation was determined using Waters DynamX 3.0. Vertical difference maps in Figures 2,3, S1 and S3 do not represent a linear sequence of non-overlapping peptides. All coincident and overlapping peptides for comparisons in each figure are provided in figure identified tabs of the Supp. Data File 3.

## Supplementary Material

Supplement 1 **Supp. Data File 1:** Supplemental figures, table and methods. PDF file containing supplemental figures S1-S6, supplemental table 1, details of NMR chemical shift assignments and BTK inhibitor analog synthesis.

Supplement 2 **Supp. Data File 2:** PDF file containing supporting NMR and MS data for acrylamide-Tirabrutinib, 2-butynamide-Ibrutinib and ‘alkane’ analog synthesis.

Supplement 3 **Supp. Data File 3:** Excel file containing details of HDX-MS experimental set-up and differences in deuterium incorporation data for all peptides. Previously published HDX MS data have been included (taken from reference (16) identified with an asterisk) and those data have the dataset identifier PXD047865 in the PRIDE partner repository.

## SIGNIFICANCE STATEMENT

Treatment of B-cell cancers such as Chronic Lymphocytic Leukemia and Mantle Cell Lymphoma has been revolutionized by the development of covalent inhibitors that target Bruton’s Tyrosine Kinase (BTK). These orally bioavailable cancer drugs are highly effective in interfering with B-cell growth and provide patients with long lasting remission. These treatments do come with vulnerabilities as inhibitor-specific resistance mutations emerge in a subset of patients. Here we investigate how chemical differences among available BTK inhibitors drive differential protein dynamics and signaling interactions that could foreshadow specific resistance mechanisms. As continuous use of BTK inhibitors progresses in time, the field will continue to learn which drugs, and which structural features of these drugs, either limit resistance or provide alternatives to established resistance.

## Abbreviations:

BTK: Bruton’s Tyrosine Kinase
PRR: proline-rich region
LKD: linker-kinase domain
BCR: B cell receptor
PLCγ: Phospholipase Cγ
HDX-MS: hydrogen/deuterium exchange-mass spectrometry
NMR: Nuclear Magnetic Resonance
WT: wild-type
DFT: Density Functional Theory
A-loop: Activation loop
