# Supplementary material for: More than an attachment module: covalent inhibitor warheads influence BTK dynamics and function
Source: bioRxiv. 2026 May 8:2026.05.07.723540. Preprint. [Version 1] doi: 10.64898/2026.05.07.723540 (PMC13174542; doi:10.64898/2026.05.07.723540)

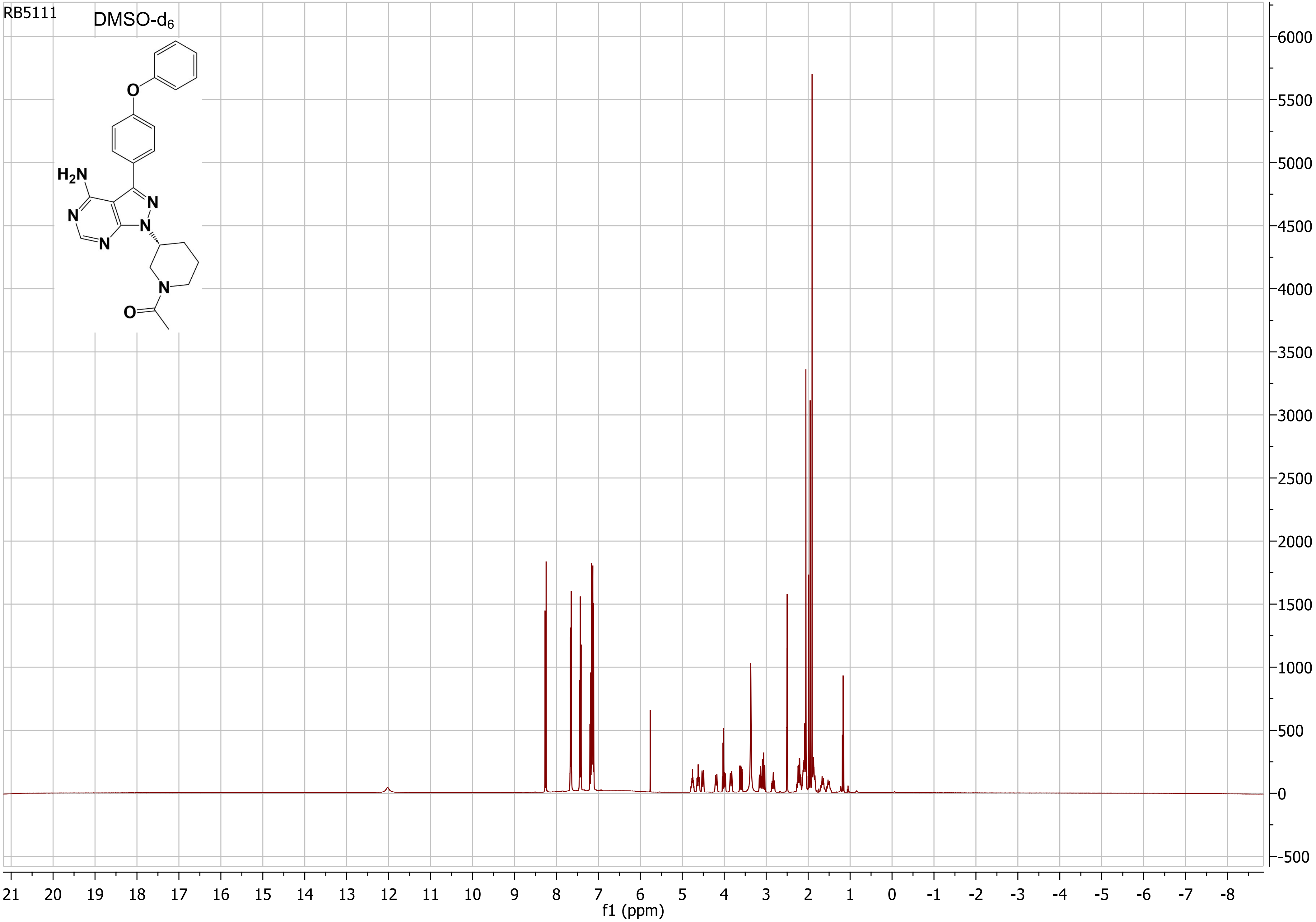

RB5111
DMSO-d6
H2N
N
N
N
N
O
N
O
6000
5500
5000
4500
4000
3500
3000
2500
2000
1500
1000
500
0
-500
21 20 19 18 17 16 15 14 13 12 11 10 9 8 7 6 5 4 3 2 1 0 -1 -2 -3 -4 -5 -6 -7 -8
f1 (ppm)

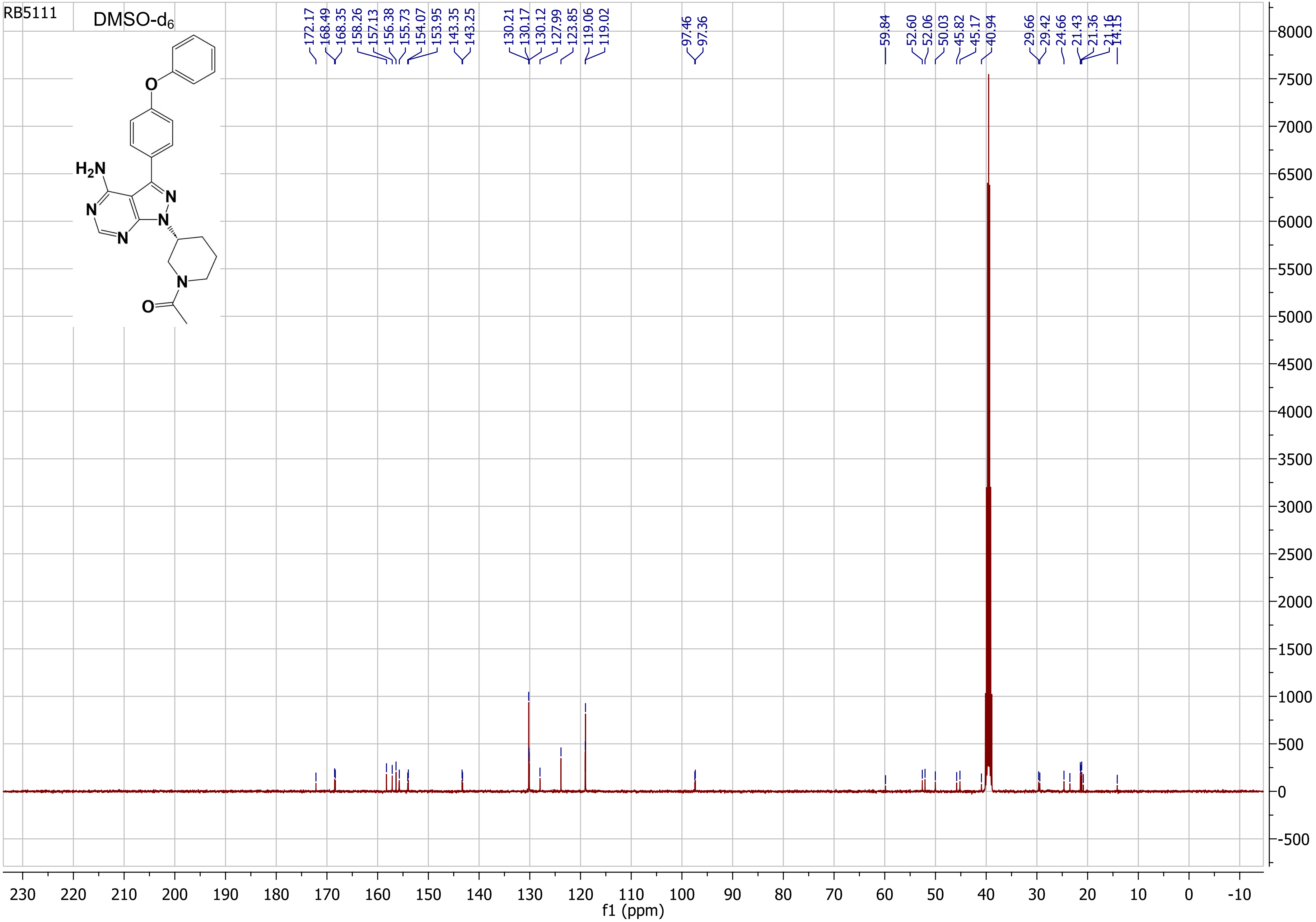

RB5111
DMSO-d6
H2N
N
N
N
N
O
N
O
172.17
168.49
168.35
158.26
157.13
156.38
155.73
154.07
153.95
143.35
143.25
130.21
130.17
130.12
127.99
123.85
119.06
119.02
97.46
97.36
59.84
52.60
52.06
50.03
45.82
45.17
40.94
29.66
29.42
24.66
21.43
21.36
21.16
14.15
f1 (ppm)

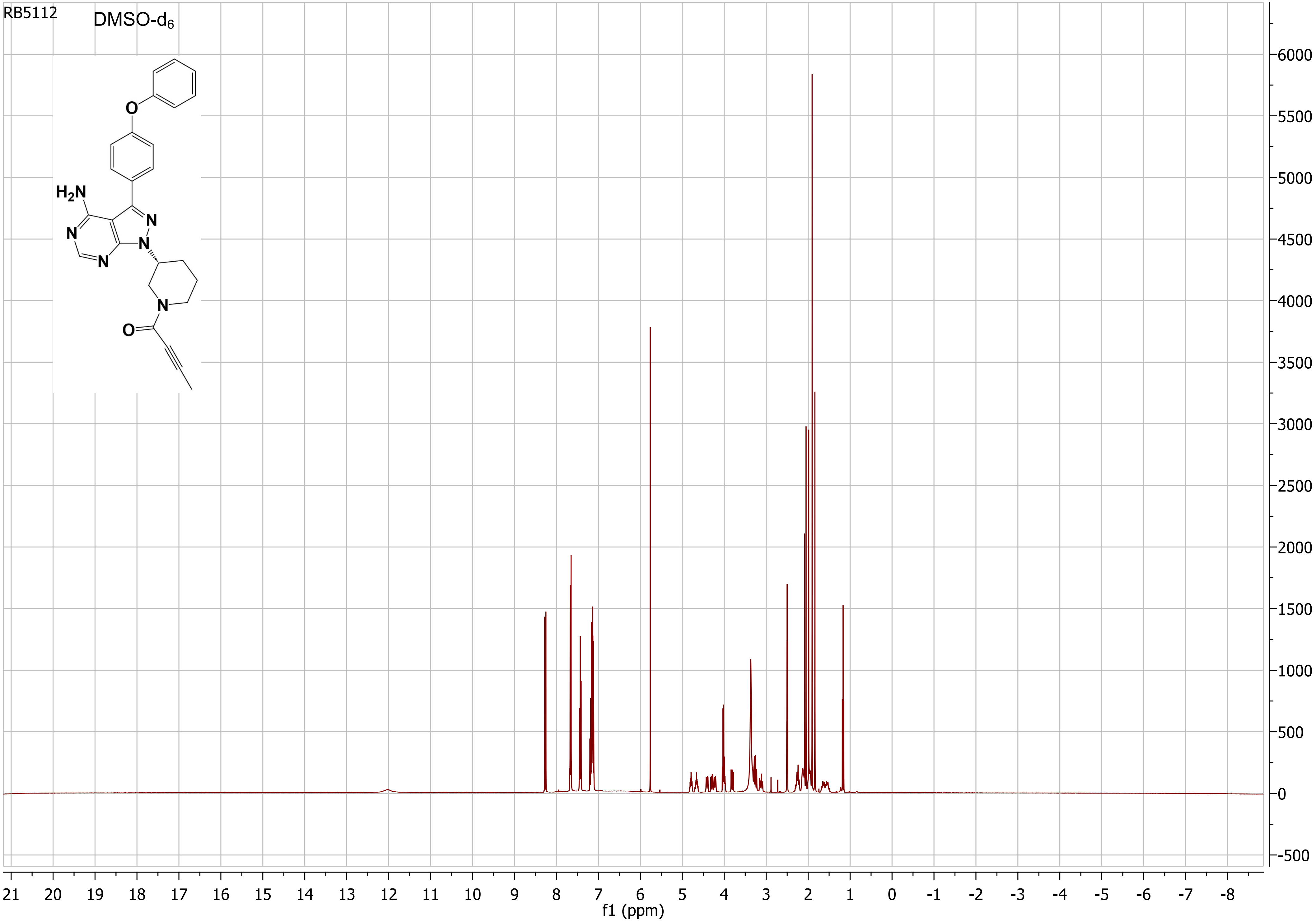
RB5112
DMSO-d6
H2N
N
N
N
N
O
N
O
21 20 19 18 17 16 15 14 13 12 11 10 9 8 7 6 5 4 3 2 1 0 -1 -2 -3 -4 -5 -6 -7 -8
f1 (ppm)
6000
5500
5000
4500
4000
3500
3000
2500
2000
1500
1000
500
0
-500

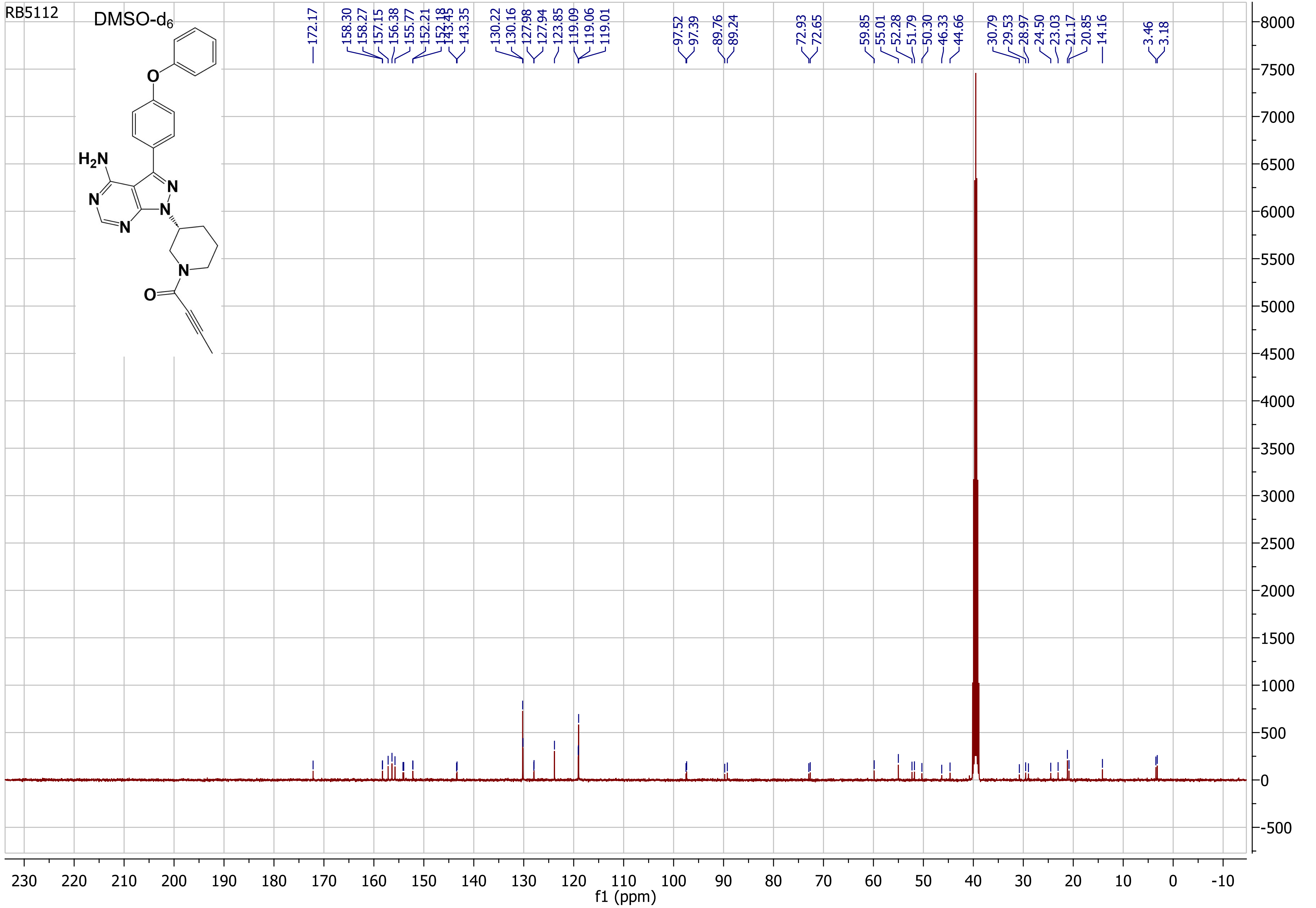
RB5112
DMSO-d6
172.17
158.30
158.27
157.15
156.38
155.77
152.21
143.35
130.22
130.16
127.98
127.94
123.85
119.09
119.06
119.01
97.52
97.39
89.76
89.24
72.93
72.65
59.85
55.01
52.28
51.79
50.30
46.33
44.66
30.79
29.53
28.97
24.50
23.03
21.17
20.85
14.16
3.46
3.18
f1 (ppm)

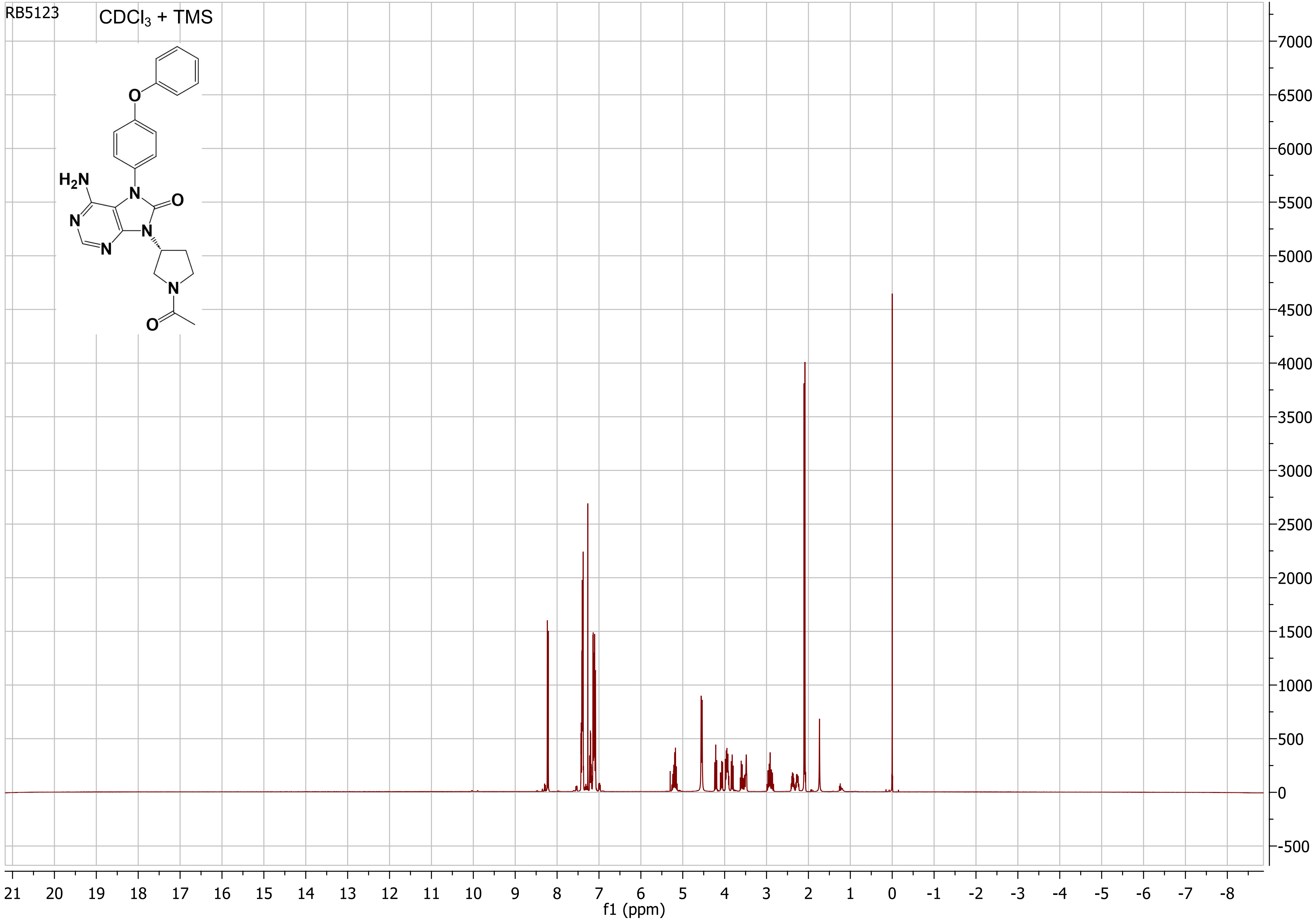
RB5123
CDCl3 + TMS
H2N
N
O
f1 (ppm)

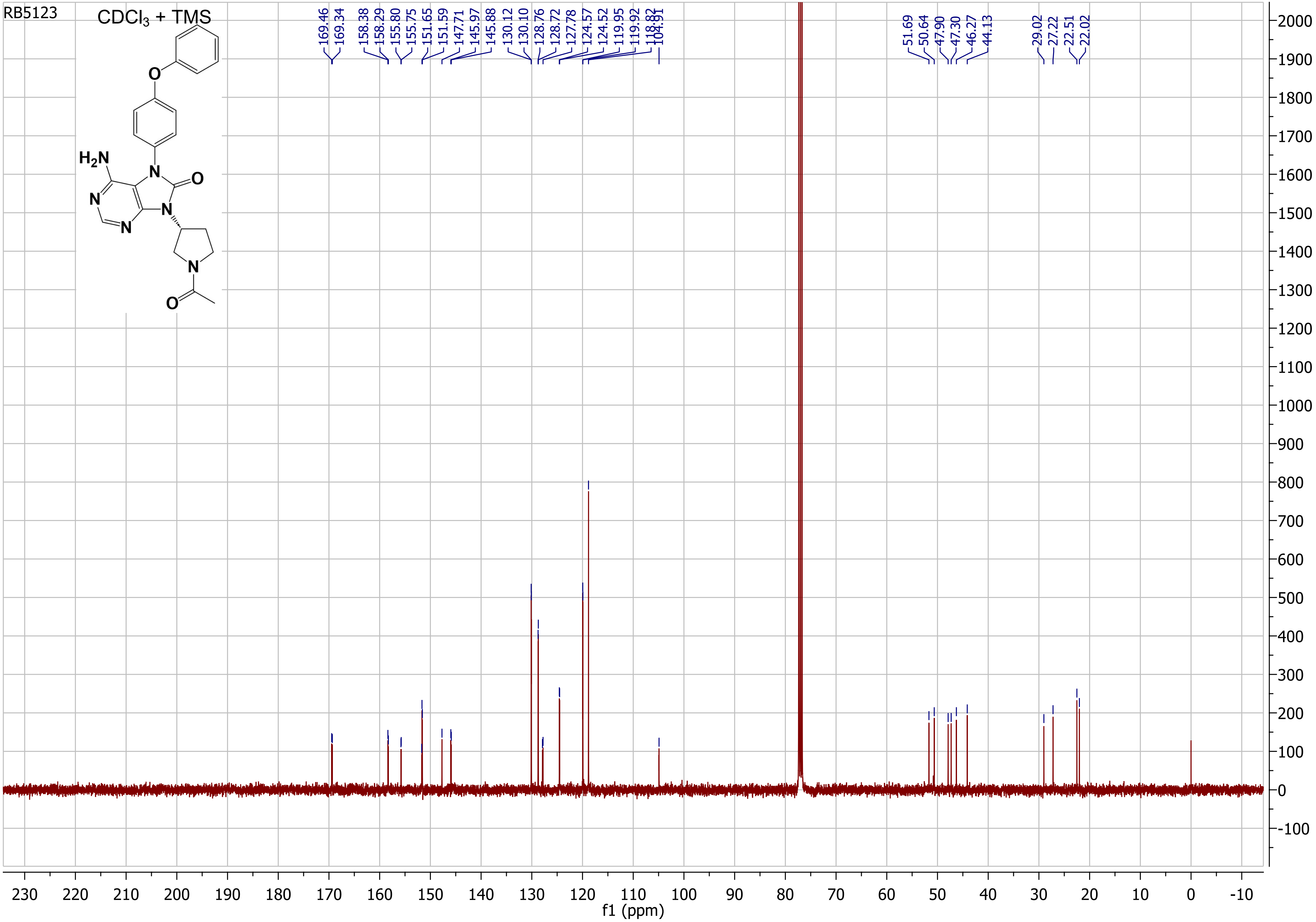
RB5123
CDCl3 + TMS
H2N
N
O
169.46
169.34
158.38
158.29
155.80
155.75
151.65
151.59
147.71
145.97
145.88
130.12
130.10
128.76
128.72
127.78
124.57
124.52
119.95
119.92
118.82
104.91
51.69
50.64
47.90
47.30
46.27
44.13
29.02
27.22
22.51
22.02
f1 (ppm)

RB5124

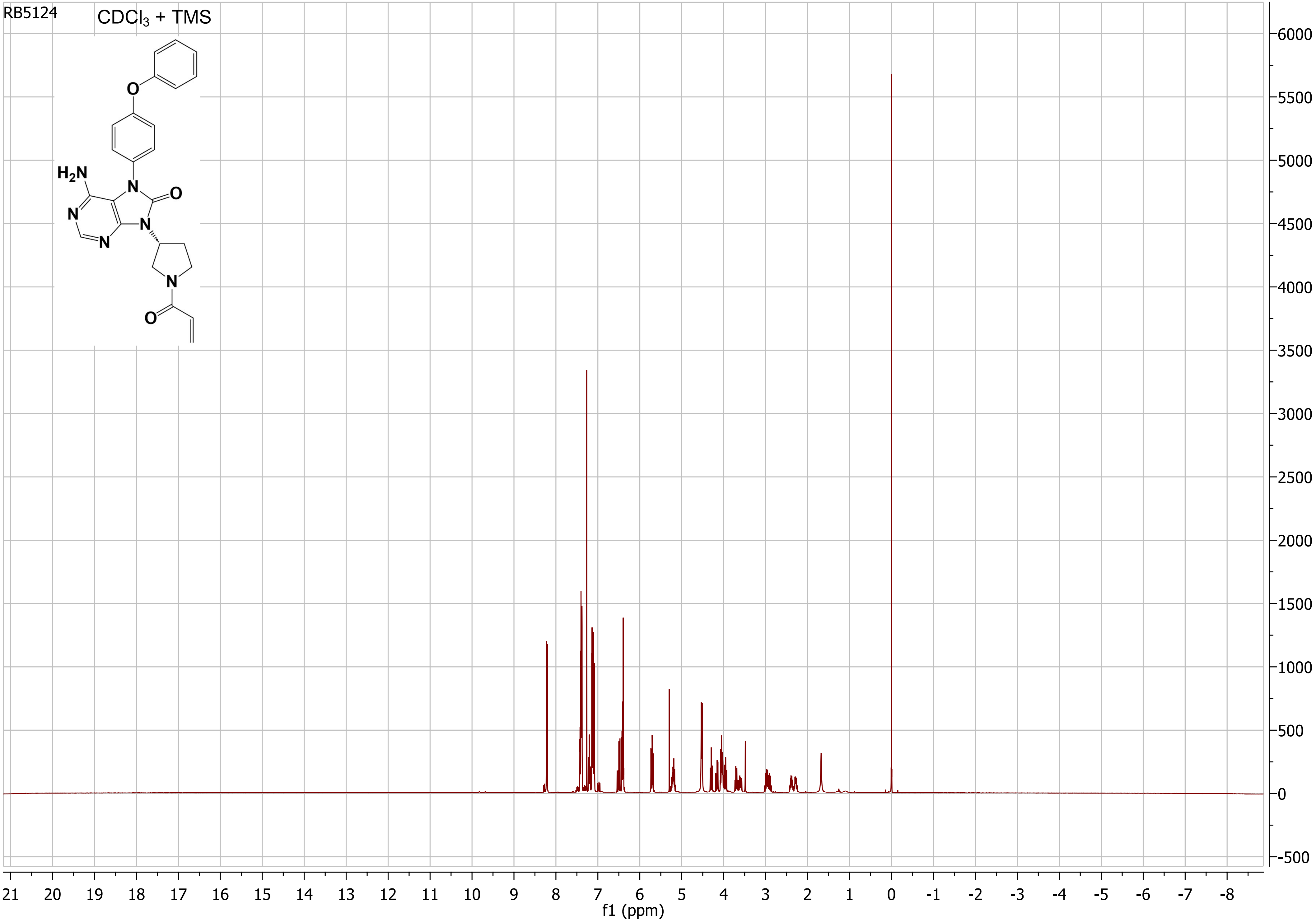

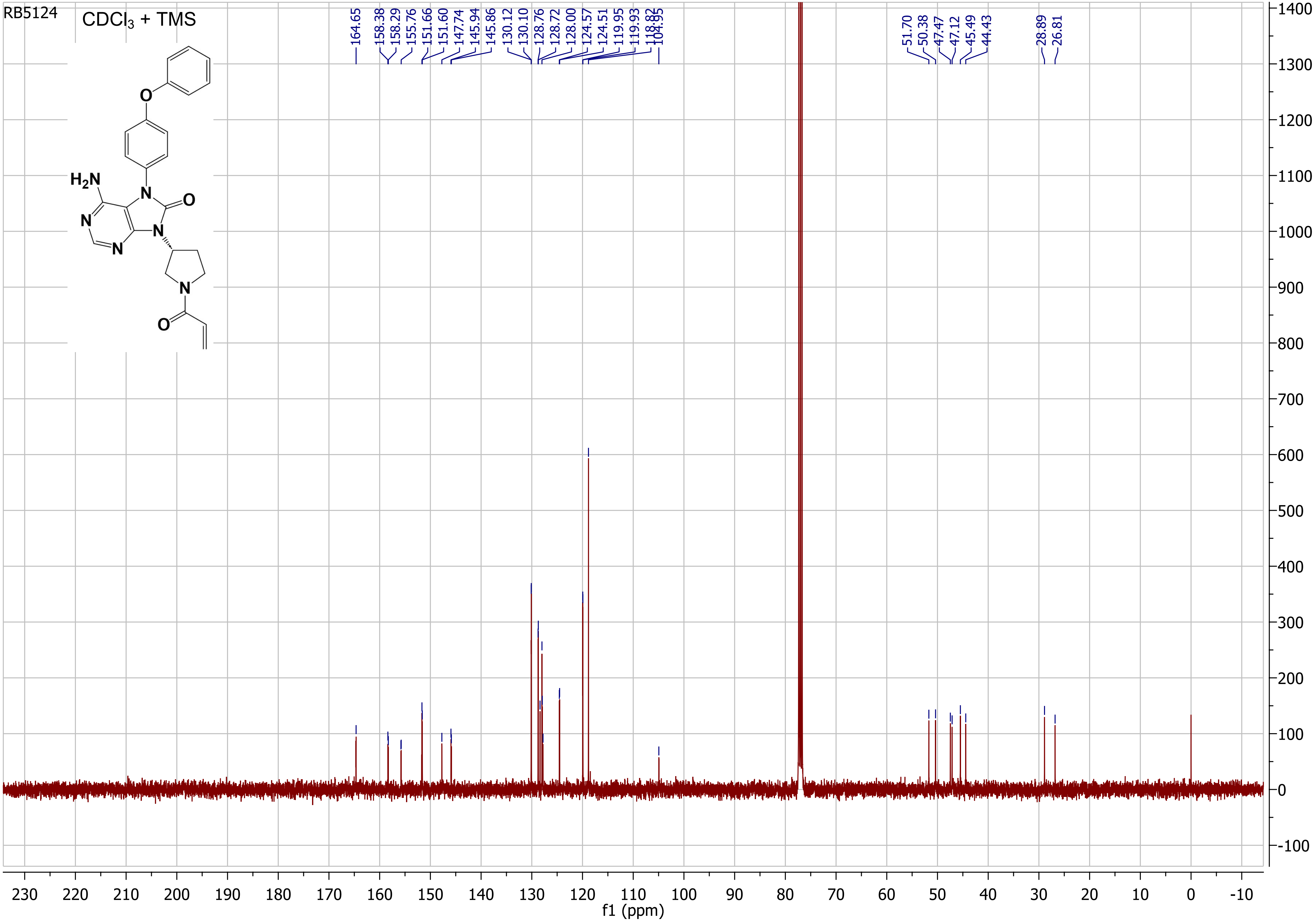

RB5124
CDCl3 + TMS
H2N
N
O
164.65
158.38
158.29
155.76
151.66
151.60
147.74
145.94
145.86
130.12
130.10
128.76
128.72
128.00
124.57
124.51
119.95
119.93
118.82
104.95
51.70
50.38
47.47
47.12
45.49
44.43
28.89
26.81
f1 (ppm)

MASS SPECTROMETRY REQUEST FORM

UNIVERSITY OF LEICESTER

DEPARTMENT OF CHEMISTRY

An Open-Access mass spectrum MUST be attached for each sample submitted otherwise samples will not be run.

Name: Rob Britton **Date:** 10/01/2023

Department/section: Chem/ Org **Tel:** 2126

Supervisor: n/a **UoL email:** rgb6@le.ac.uk

Sample Name:	Solvent Used:	UV Wave length for LCMS
RB5111	MeOH	260

Full Molecular Structure (insert image below)

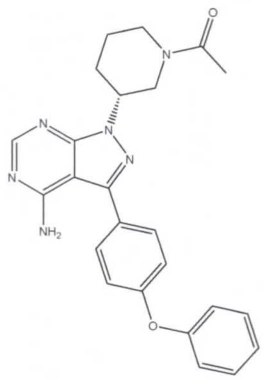

additional sample text (optional)

Molecular Formula (eg Cxx Hyy Nz *etc.*)	Molecular Weight
C24 H24 N6 O2	428.50

Any specific sample information *(optional)*

click to enter here

Safety & handling Information: Is your compound Toxic, Hydroscopic *etc.*?

click to enter here

Analysis Request - TOF Accurate Mass

**ESI/LCMS:**	☒	**MS/MS:**	☐ Please discuss
**ASAP:** Atmopheric Solids Analysis Probe	☐	**Custom Exp:**	☐ Please discuss
**APCI/LCMS:**	☐	**Simulation:**	☐

Operator Use Only

Date Run	Data Stored as:	Technique	Results
12/ 01 /23	G 2 # 4874	ESI+	$[MH]^+$ 429 ✓
/ /	G #		$[MNa]^+$ 451 ✓
/ /	G #		
/ /	G #		

Comments:

***Please contact Sharad Mistry (scm11@) if you require any further help or advice**

ID:Research-90-2
Time:15:08:23
Vial:2:37

Description:RB5111
Method:D:\OA Methods\2_Scan_150_650_Pos-Neg.olp
UserName:Research-

Date:10-Jan-2023

1:(Time: 0.44) Combine (167:209-(27:70+827:870)) 1:MS ES+ 1.1e+006

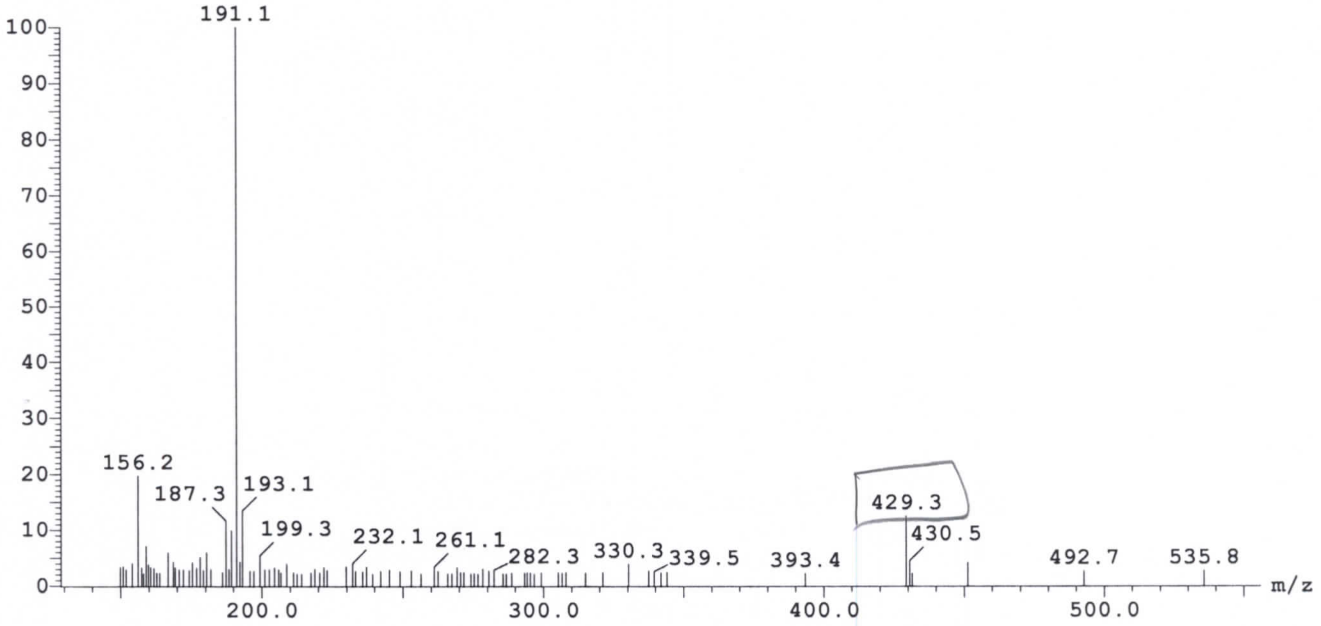

1:(Time: 0.45) Combine (170:212-(25:68+1000:1043)) 2:MS ES- 1.6e+004

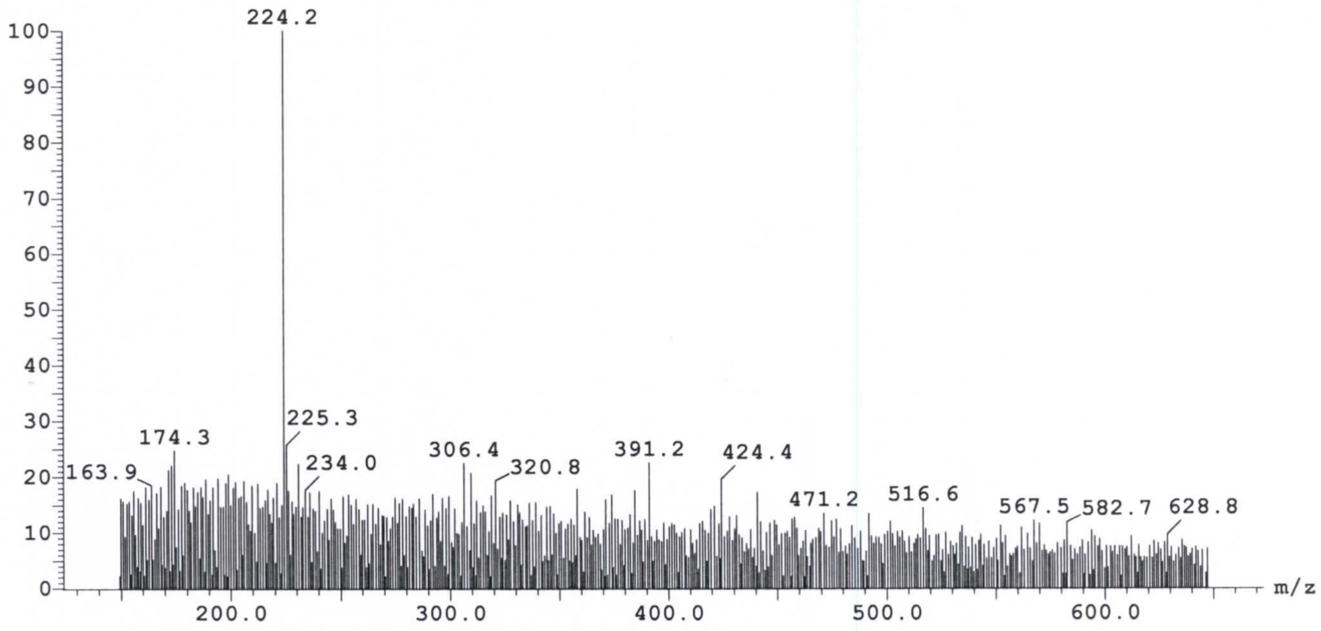

RB5111

12-Jan-2023
12:24:24

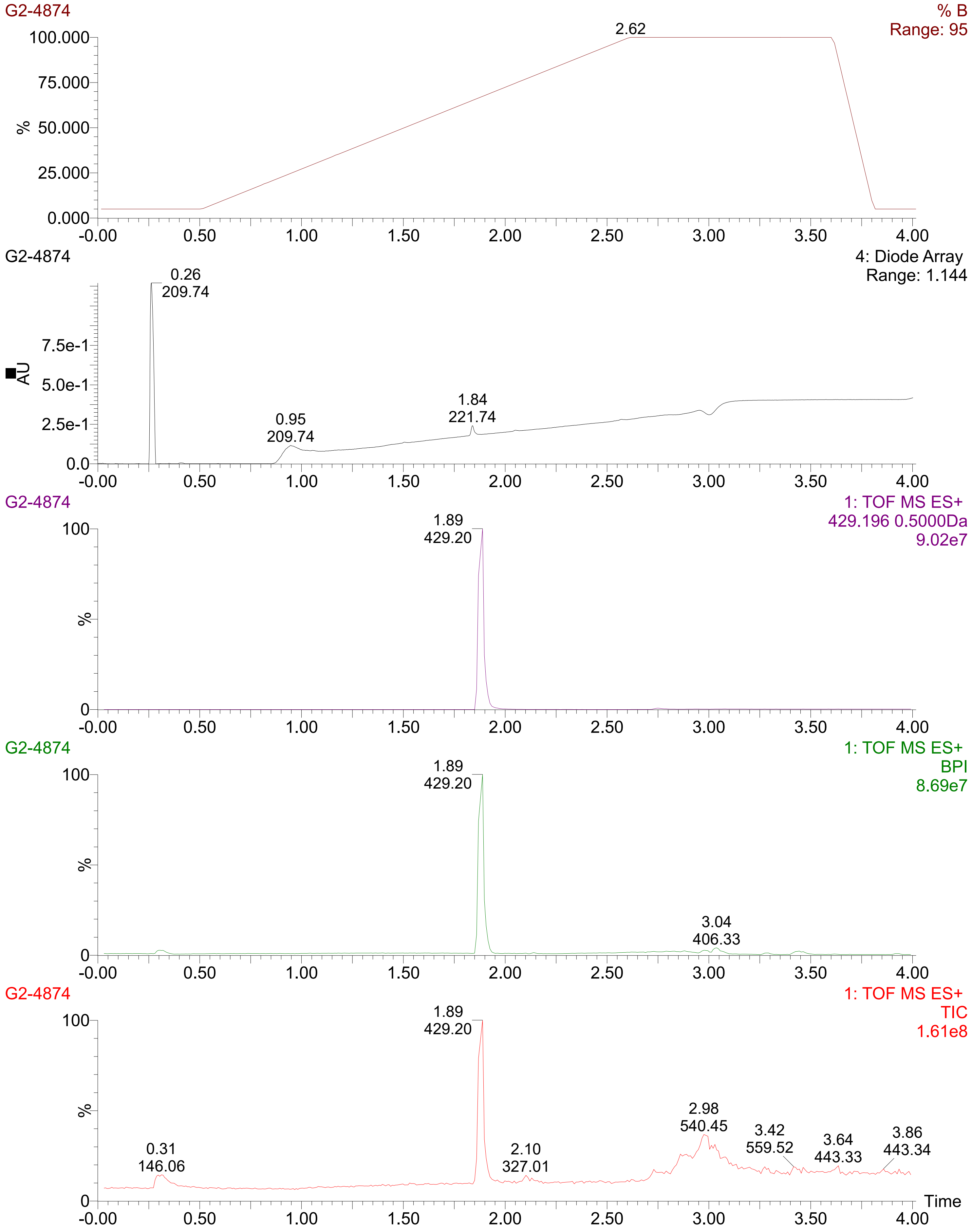

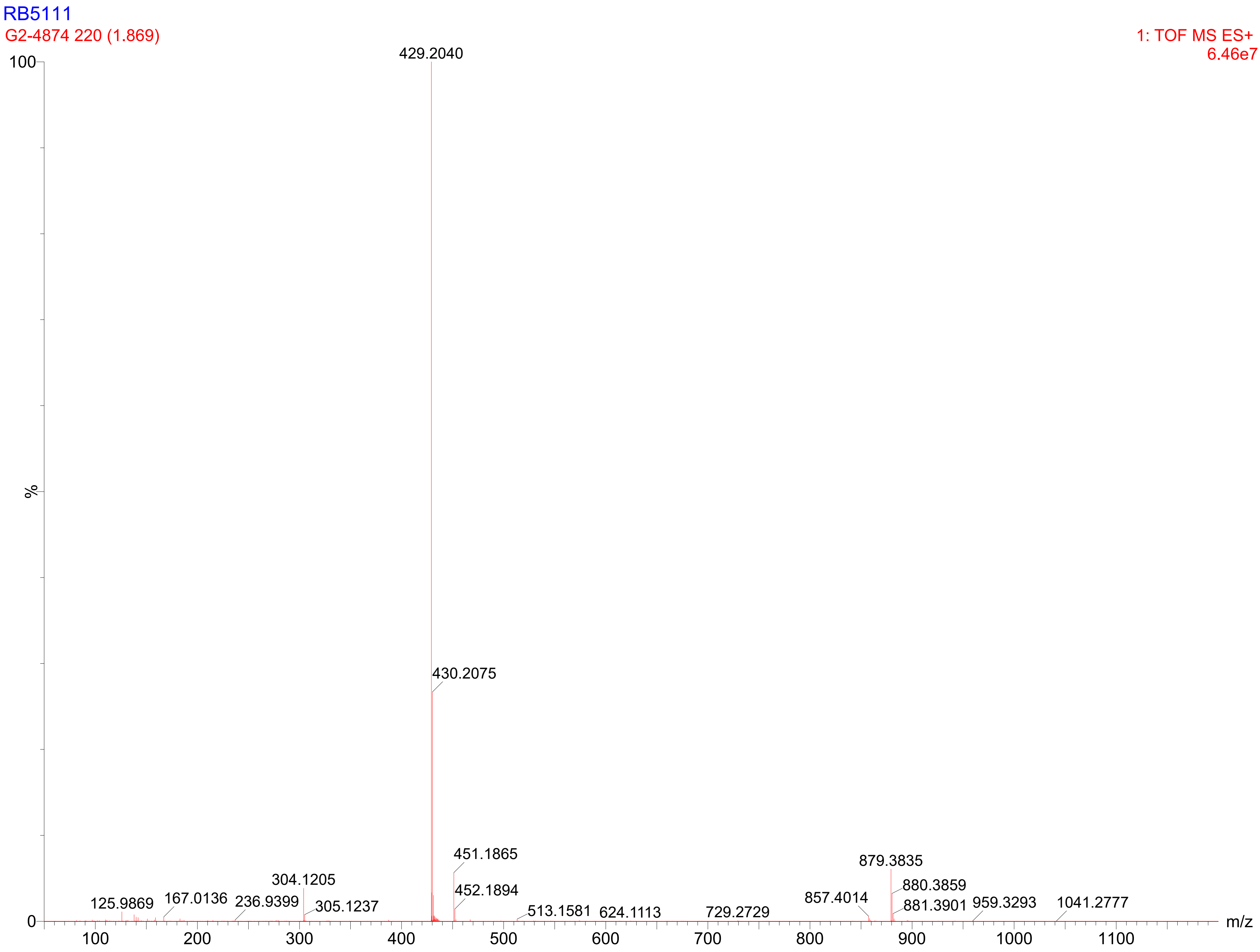

RB5111
G2-4874 220 (1.869)
1: TOF MS ES+
6.46e7
429.2040
430.2075
451.1865
452.1894
304.1205
305.1237
125.9869
167.0136
236.9399
513.1581
624.1113
729.2729
857.4014
879.3835
880.3859
881.3901
959.3293
1041.2777
100
0
%
m/z
100
200
300
400
500
600
700
800
900
1000
1100

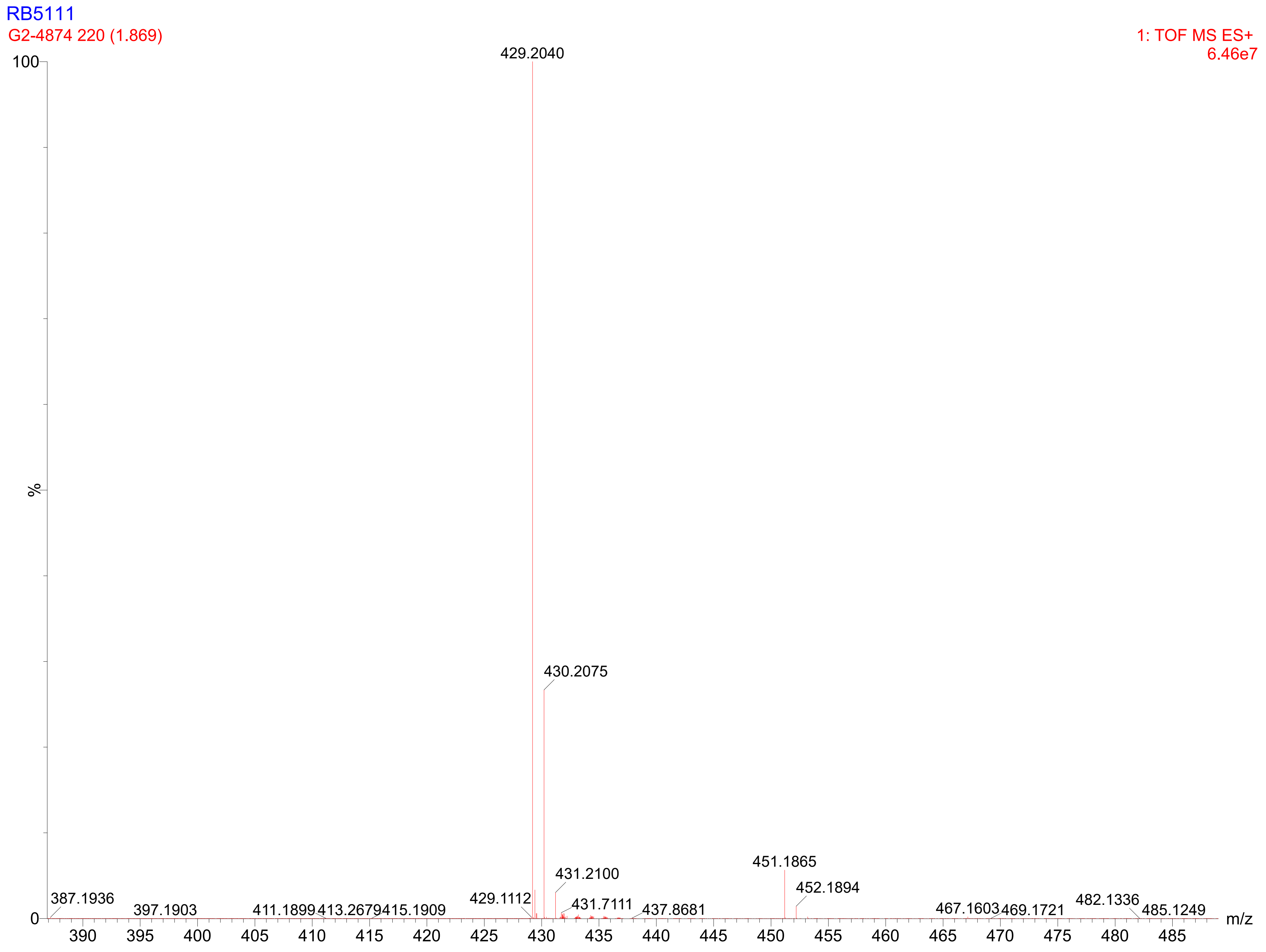
RB5111
G2-4874 220 (1.869)
1: TOF MS ES+
6.46e7
100
%
0
429.2040
430.2075
431.2100
431.7111
437.8681
451.1865
452.1894
387.1936
397.1903
411.1899
413.2679
415.1909
429.1112
467.1603
469.1721
482.1336
485.1249
390
395
400
405
410
415
420
425
430
435
440
445
450
455
460
465
470
475
480
485
m/z

Elemental Composition Report [MH]+

Single Mass Analysis

Tolerance = 5.0 PPM / DBE: min = -1.5, max = 100.0

Element prediction: Off

Number of isotope peaks used for i-FIT = 3

Monoisotopic Mass, Even Electron Ions

437 formula(e) evaluated with 1 results within limits (up to 100 closest results for each mass)

Elements Used:

C: 24-24 H: 0-150 N: 0-30 O: 0-30

Minimum:				-1.5				
Maximum:		5.0	5.0	100.0				
Mass	Calc. Mass	mDa	PPM	DBE	i-FIT	Norm	Conf(%)	Formula
429.2040	429.2039	0.1	0.2	15.5	889.4	n/a	n/a	C24 H25 N6 O2

Elemental Composition Report [MNa]+

Single Mass Analysis

Tolerance = 5.0 PPM / DBE: min = -1.5, max = 100.0

Element prediction: Off

Number of isotope peaks used for i-FIT = 3

Monoisotopic Mass, Even Electron Ions

914 formula(e) evaluated with 1 results within limits (up to 100 closest results for each mass)

Elements Used:

C: 24-24 H: 0-150 N: 0-30 O: 0-30 Na: 0-1

Minimum:				-1.5				
Maximum:		5.0	5.0	100.0				
Mass	Calc. Mass	mDa	PPM	DBE	i-FIT	Norm	Conf(%)	Formula
451.1865	451.1858	0.7	1.6	15.5	409.4	n/a	n/a	C24 H24 N6 O2 Na

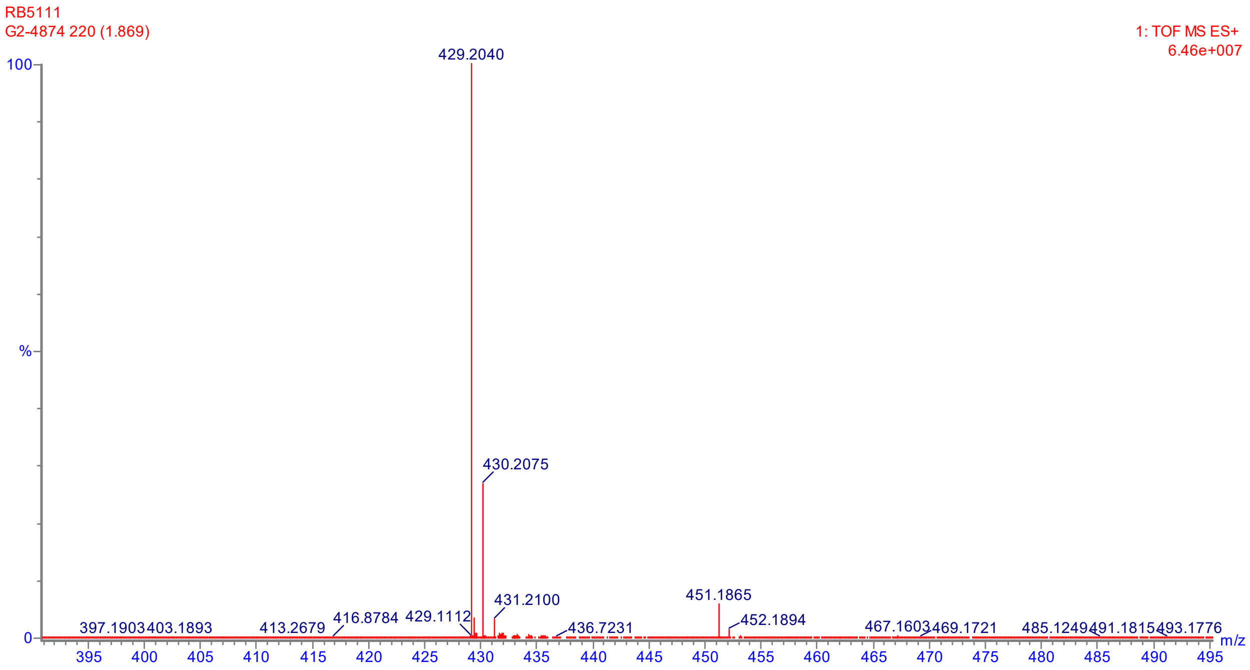

UNIVERSITY OF LEICESTER

MASS SPECTROMETRY REQUEST FORM

DEPARTMENT OF CHEMISTRY

An Open-Access mass spectrum MUST be attached for each sample submitted otherwise samples will not be run.

Name: Rob Britton
Date: 10/01/2023
Department/section: Chem/ Org
Tel: 2126
Supervisor: n/a
UoL email: rgb6@le.ac.uk

Sample Name:	Solvent Used:	UV Wave length for LCMS
RB5112	MeOH	260

Full Molecular Structure (insert image below)

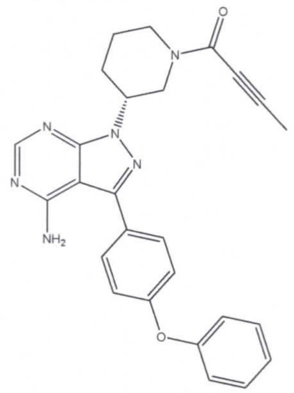

additional sample text (optional)

Molecular Formula (eg Cxx Hyy Nz *etc.*)	Molecular Weight
C26 H24 N6 O2	452.52

Any specific sample information *(optional)*

click to enter here

Safety & handling Information: Is your compound Toxic, Hydroscopic *etc.*?

click to enter here

Analysis Request - TOF Accurate Mass

- **ESI/LCMS:** ☒
- **ASAP:** ☐ Atmopheric Solids Analvsis Probe
- **APCI/LCMS:** ☐
- **MS/MS:** ☐ Please discuss
- **Custom Exp:** ☐ Please discuss
- **Simulation:** ☐

Operator Use Only

Date Run	Data Stored as:	Technique	Results
12/ 01 /23	G 2 # 4875	ESI+	$[MH]^+$ 453 ✓
/ /	G #		$[MNa]^+$ 475 ✓
/ /	G #		
/ /	G #		

Comments:

***Please contact Sharad Mistry (scm11@) if you require any further help or advice**

Openlynx Report - Research-

ID:Research-90-4
Time:15:16:30
Vial:2:39

Description:RB5112
Method:D:\OA Methods\2_Scan_150_650_Pos-Neg.olp
UserName:Research-

Date:10-Jan-2023

1: (Time: 0.43) Combine (165:207-(12:55+826:869))

1:MS ES+
9.7e+005

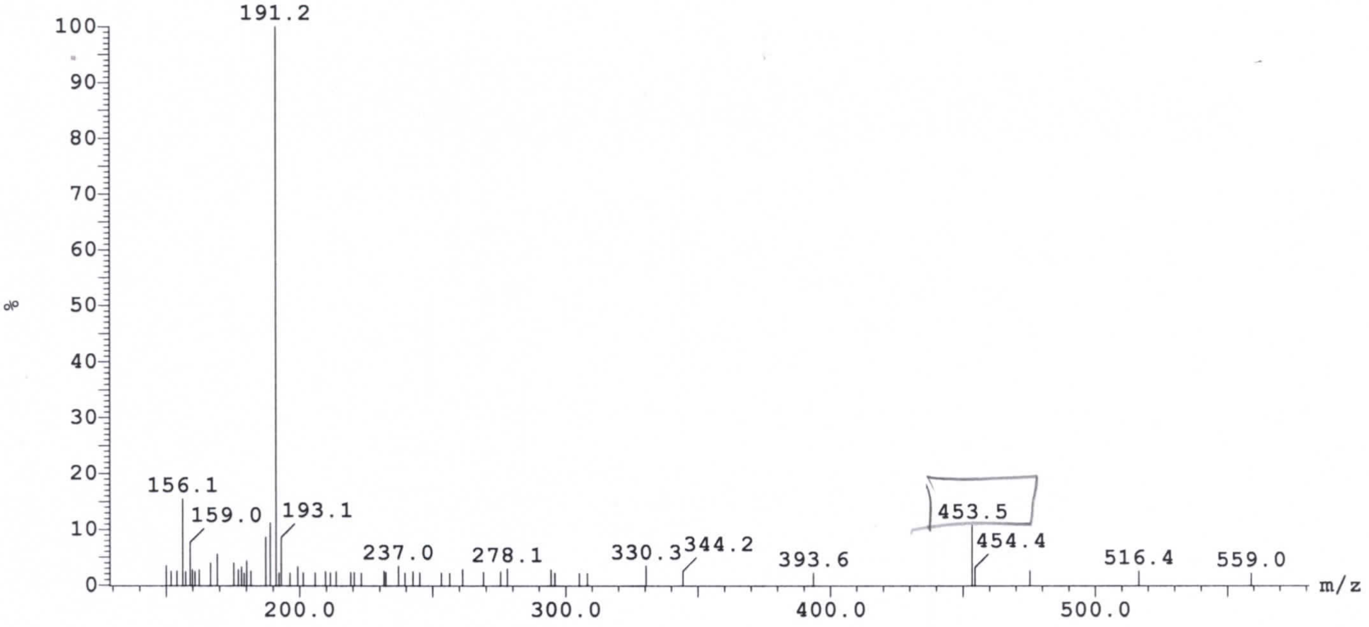

1: (Time: 0.43) Combine (162:204-(5:48+985:1028))

2:MS ES-
1.1e+004

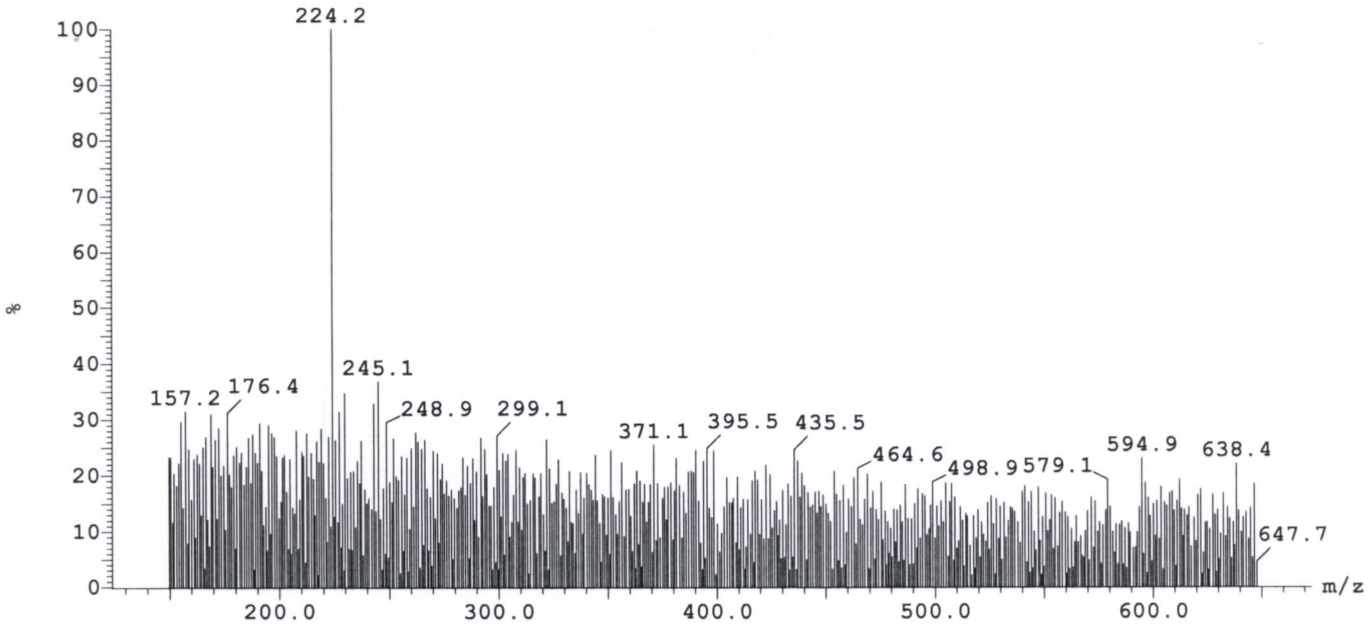

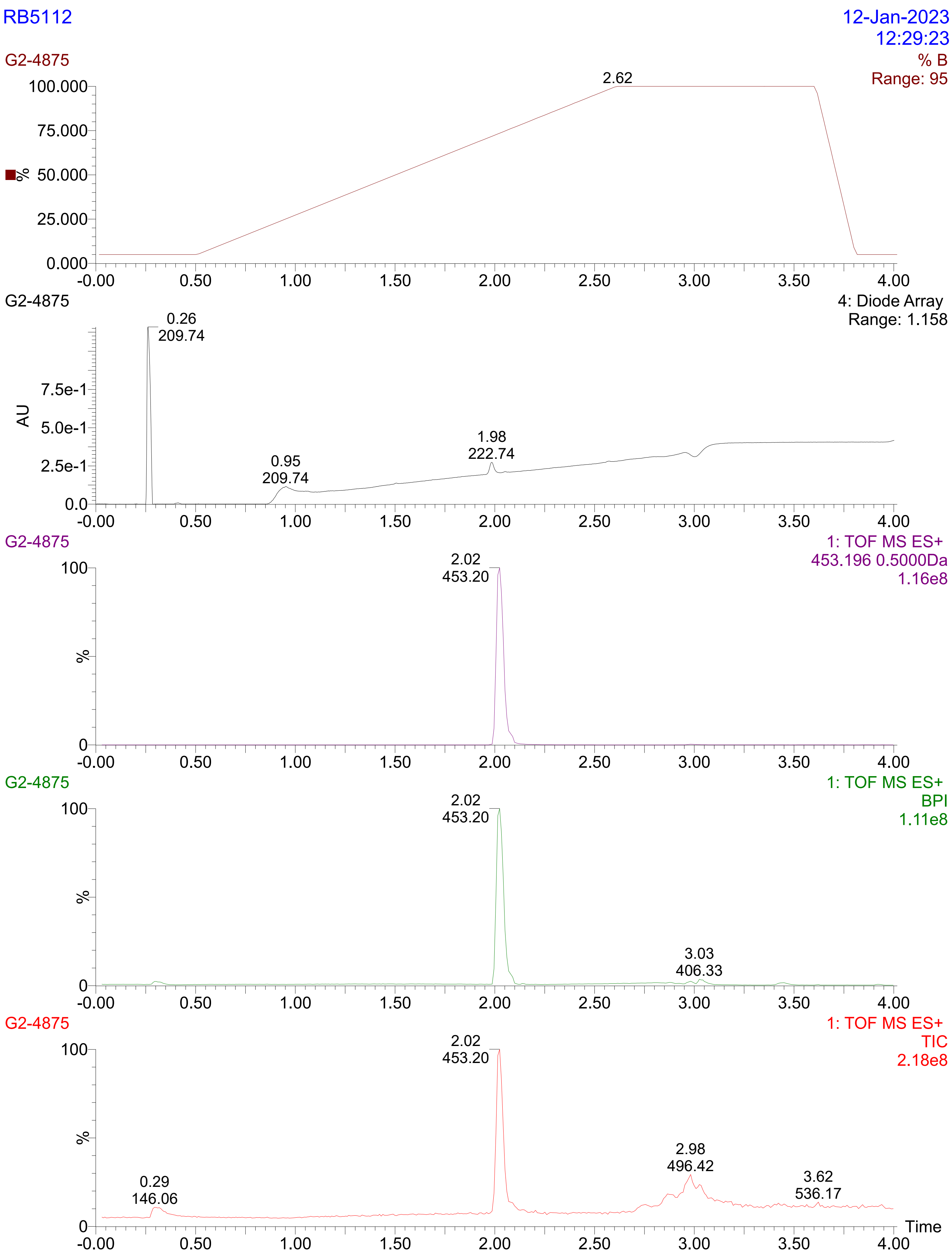

RB5112
12-Jan-2023
12:29:23
G2-4875
% B
Range: 95
2.62
100.000
75.000
%
50.000
25.000
0.000
-0.00
0.50
1.00
1.50
2.00
2.50
3.00
3.50
4.00
G2-4875
4: Diode Array
Range: 1.158
0.26
209.74
7.5e-1
AU
5.0e-1
2.5e-1
0.0
0.95
209.74
1.98
222.74
G2-4875
1: TOF MS ES+
453.196 0.5000Da
1.16e8
2.02
453.20
100
%
0
G2-4875
1: TOF MS ES+
BPI
1.11e8
2.02
453.20
3.03
406.33
G2-4875
1: TOF MS ES+
TIC
2.18e8
2.02
453.20
0.29
146.06
2.98
496.42
3.62
536.17
Time

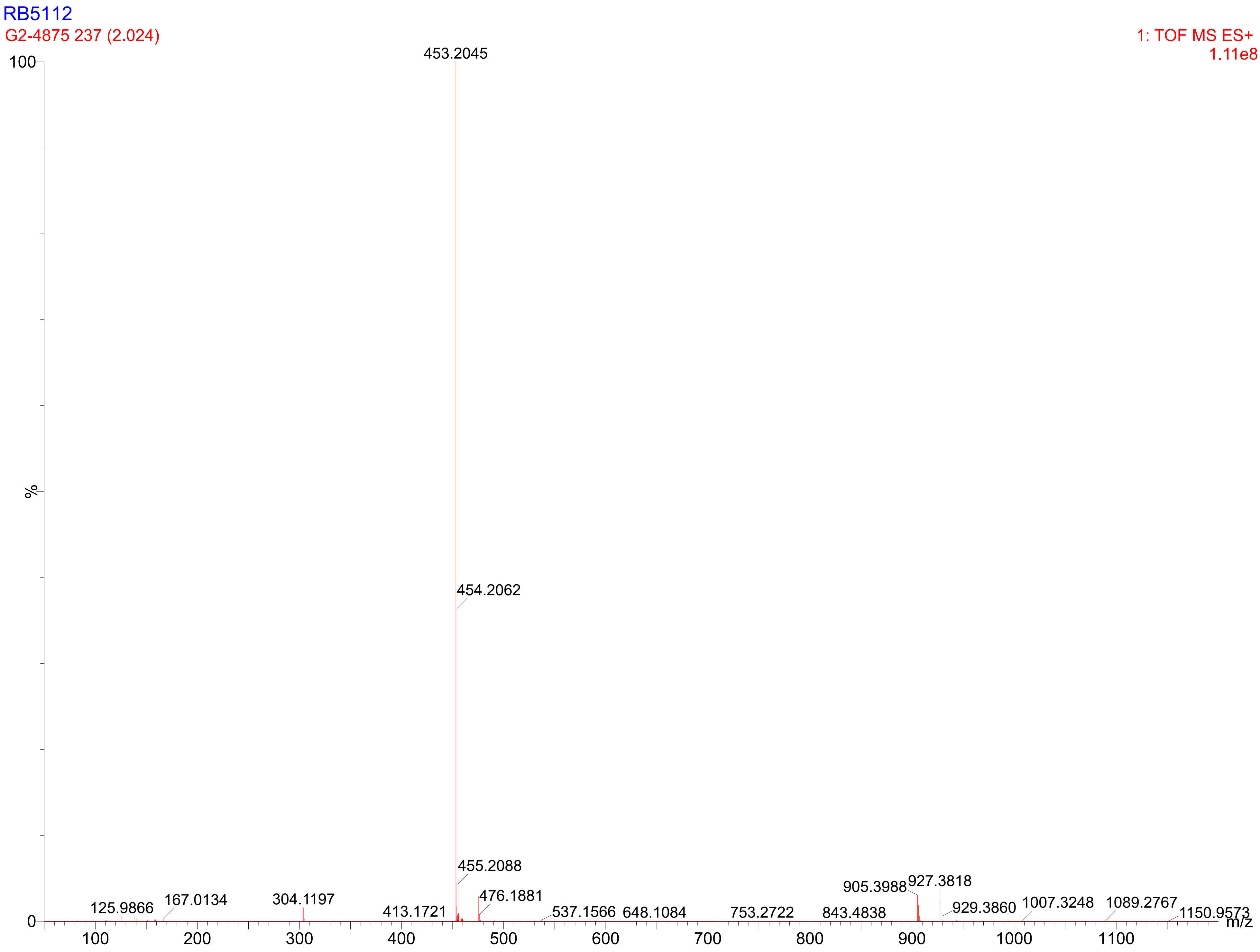
RB5112
G2-4875 237 (2.024)
1: TOF MS ES+
1.11e8
453.2045
454.2062
455.2088
476.1881
125.9866
167.0134
304.1197
413.1721
537.1566
648.1084
753.2722
843.4838
905.3988
927.3818
929.3860
1007.3248
1089.2767
1150.9573
100
%
0
m/z
100
200
300
400
500
600
700
800
900
1000
1100

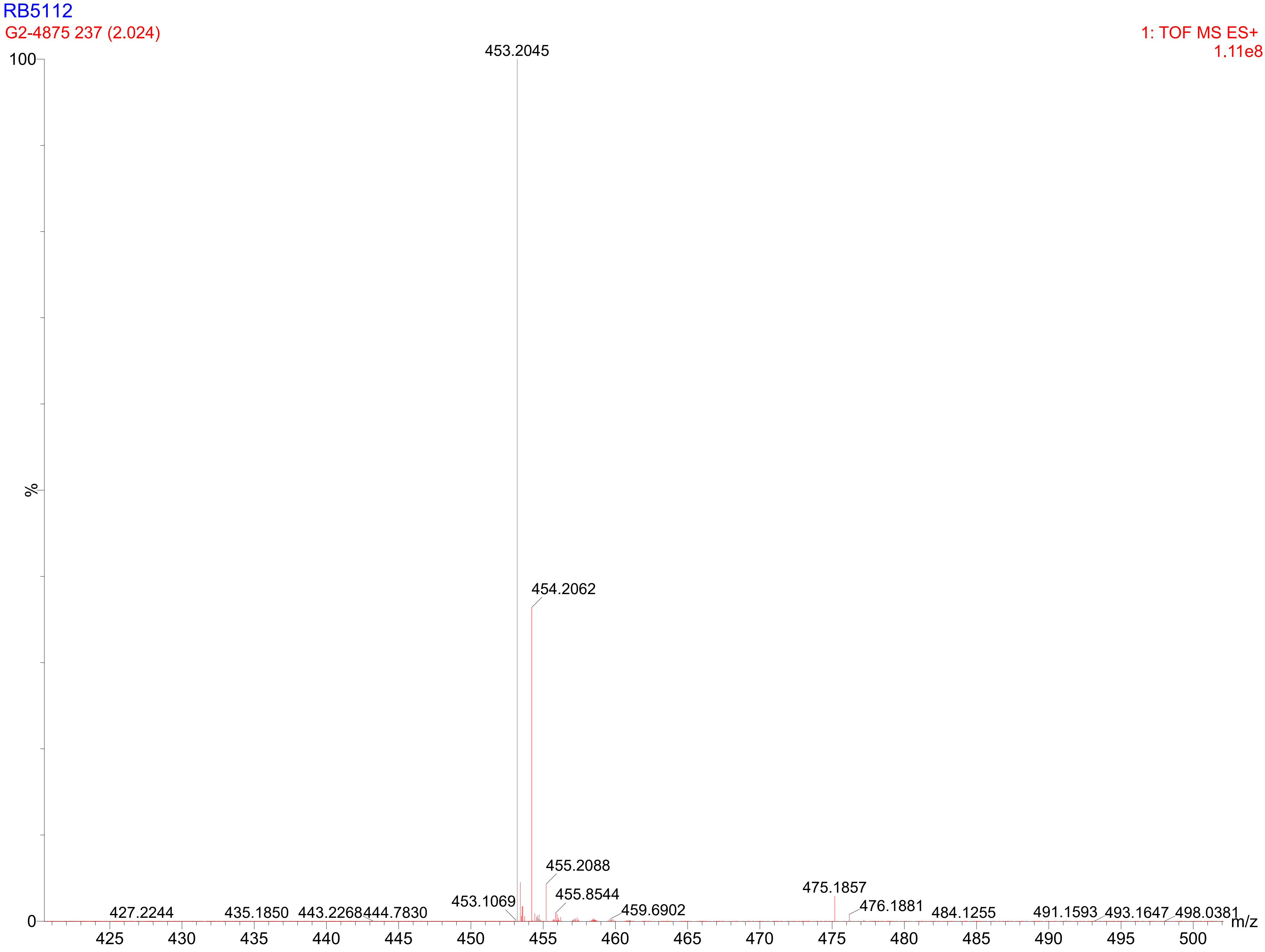
RB5112
G2-4875 237 (2.024)
1: TOF MS ES+
1.11e8
100
%
0
453.2045
454.2062
455.2088
455.8544
453.1069
459.6902
475.1857
476.1881
427.2244
435.1850
443.2268
444.7830
484.1255
491.1593
493.1647
498.0381
m/z
425
430
435
440
445
450
455
460
465
470
475
480
485
490
495
500

Elemental Composition Report [MH]+

Single Mass Analysis
Tolerance = 5.0 PPM / DBE: min = -1.5, max = 100.0
Element prediction: Off
Number of isotope peaks used for i-FIT = 3
Monoisotopic Mass, Even Electron Ions
483 formula(e) evaluated with 1 results within limits (up to 100 closest results for each mass)
Elements Used:
C: 26-26 H: 0-150 N: 0-30 O: 0-30

Minimum:				-1.5				
Maximum:		5.0	5.0	100.0				
Mass	Calc. Mass	mDa	PPM	DBE	i-FIT	Norm	Conf(%)	Formula
453.2045	453.2039	0.6	1.3	17.5	941.8	n/a	n/a	C26 H25 N6 O2

Elemental Composition Report [MNa]+

Single Mass Analysis
Tolerance = 5.0 PPM / DBE: min = -1.5, max = 100.0
Element prediction: Off
Number of isotope peaks used for i-FIT = 3
Monoisotopic Mass, Even Electron Ions
1007 formula(e) evaluated with 1 results within limits (up to 100 closest results for each mass)
Elements Used:
C: 26-26 H: 0-150 N: 0-30 O: 0-30 Na: 0-1

Minimum:				-1.5				
Maximum:		5.0	5.0	100.0				
Mass	Calc. Mass	mDa	PPM	DBE	i-FIT	Norm	Conf(%)	Formula
475.1857	475.1858	-0.1	-0.2	17.5	348.5	n/a	n/a	C26 H24 N6 O2 Na

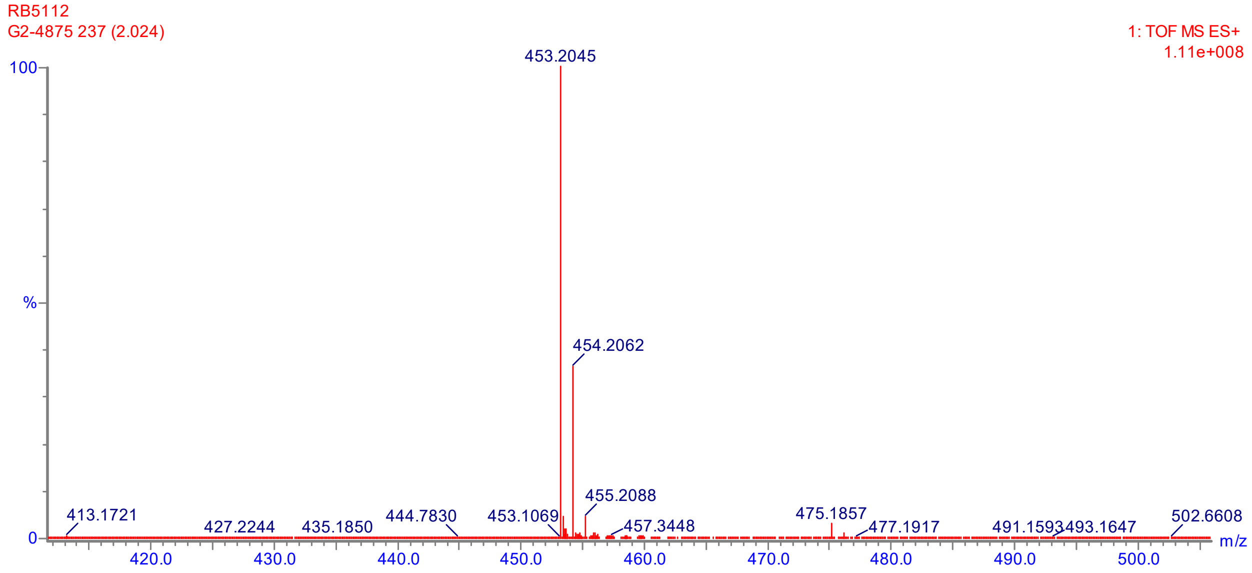

UNIVERSITY OF LEICESTER

MASS SPECTROMETRY REQUEST FORM

DEPARTMENT OF CHEMISTRY

An Open-Access mass spectrum MUST be attached for each sample submitted otherwise samples will not be run.

Name: Rob Britton

Date: 17/06/2024

Department/section: Chem

Tel: click to enter here

Supervisor: Click to enter here

UoL email: rgb6@le.ac.uk

Sample Name:	Solvent Used:	UV Wave length for LCMS
RB5123	MeOH	215

Full Molecular Structure (insert image below)

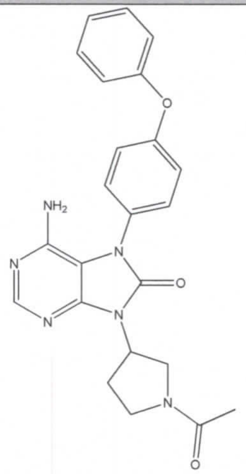

additional sample text (optional)

**Molecular Formula** (eg Cxx Hyy Nz *etc.*)	**Molecular Weight**
C23H22N6O3	430.47

Any specific sample information *(optional)*

click to enter here

Safety & handling Information: Is your compound Toxic, Hydroscopic *etc.*?

n/a

Analysis Request - TOF Accurate Mass

**ESI/LCMS:**	☒	**MS/MS:**	☐ Please discuss
**ASAP:** Atmopheric Solids Analysis Probe	☐	**Custom Exp:**	☐ Please discuss
**APCI/LCMS:**	☐	**Simulation:**	☐

Operator Use Only

Date Run	Data Stored as:	Technique	Results
17/ 06 /24	G 2# 6264	ESI+	$[MH]^+$ 431 ✓
/ /	G #		
/ /	G #		
/ /	G #		

Comments:

*Please contact Sharad Mistry (scm11@) if you require any further help or advice

1: (Time: 0.32) Combine (116:158-(1:32+773:816))

1:MS ES+
7.0e+005

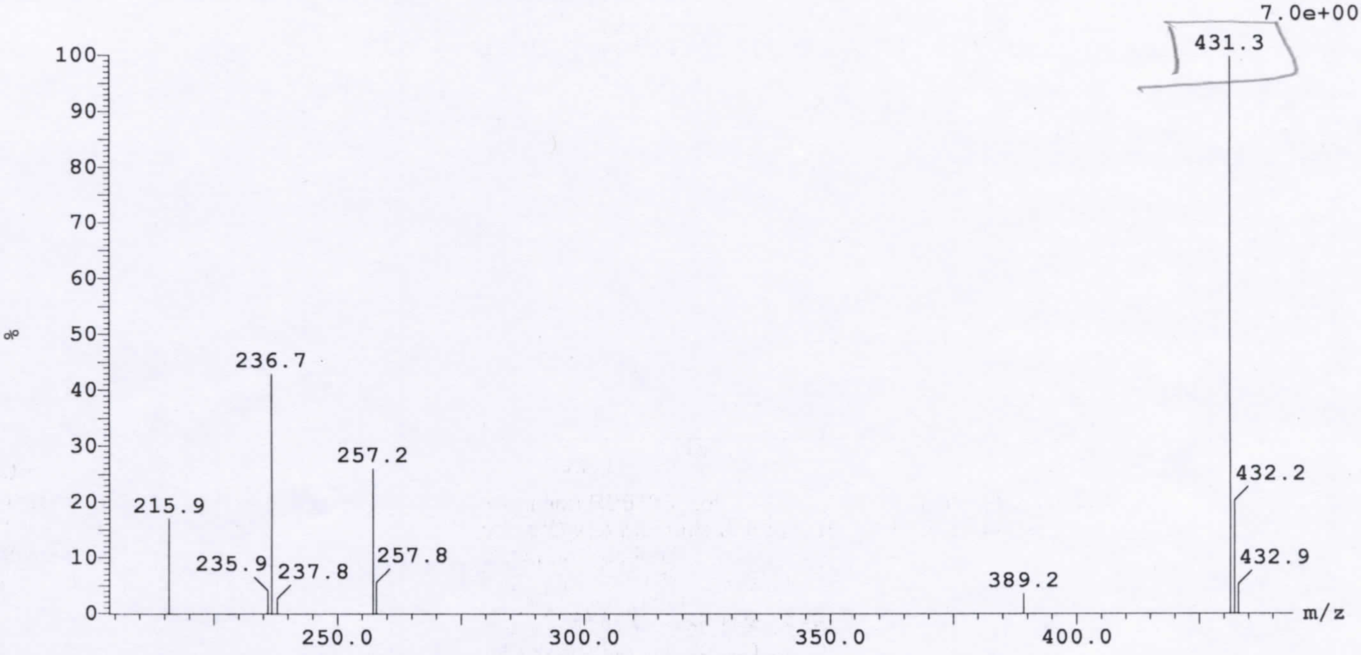

2: (Time: 0.69) Combine (273:315-(190:233+924:967))

2:MS ES-
2.2e+003

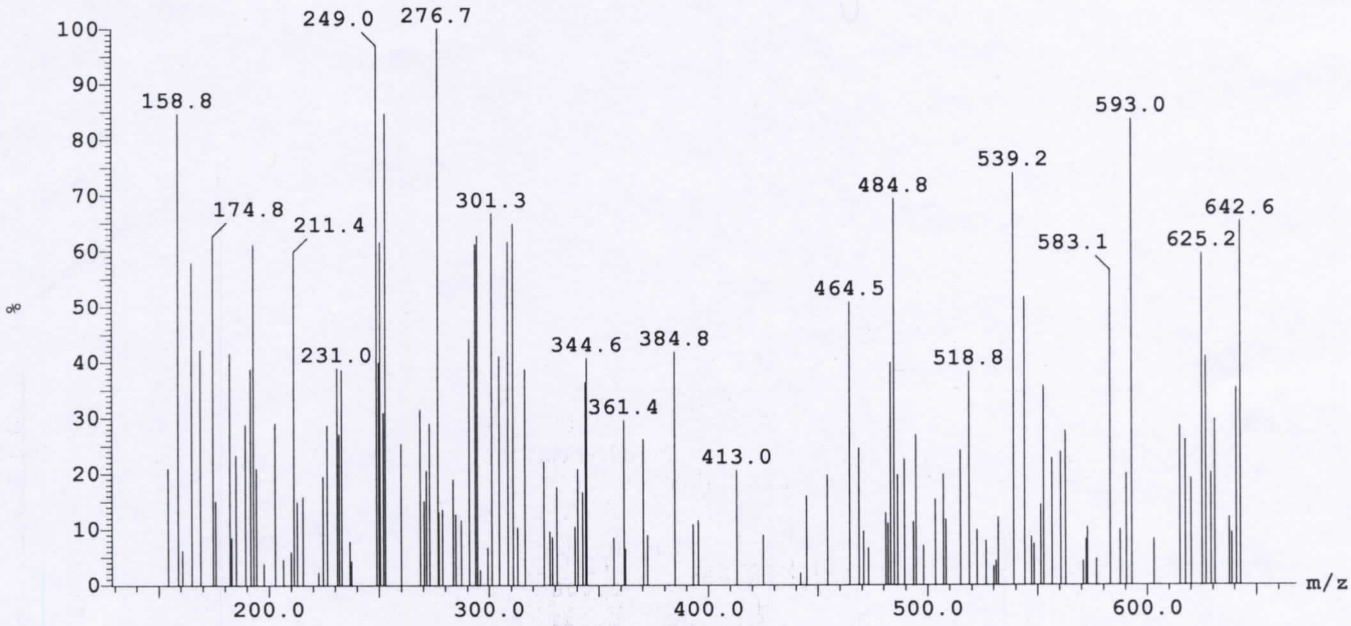

RB5123

17-Jun-2024
13:41:36

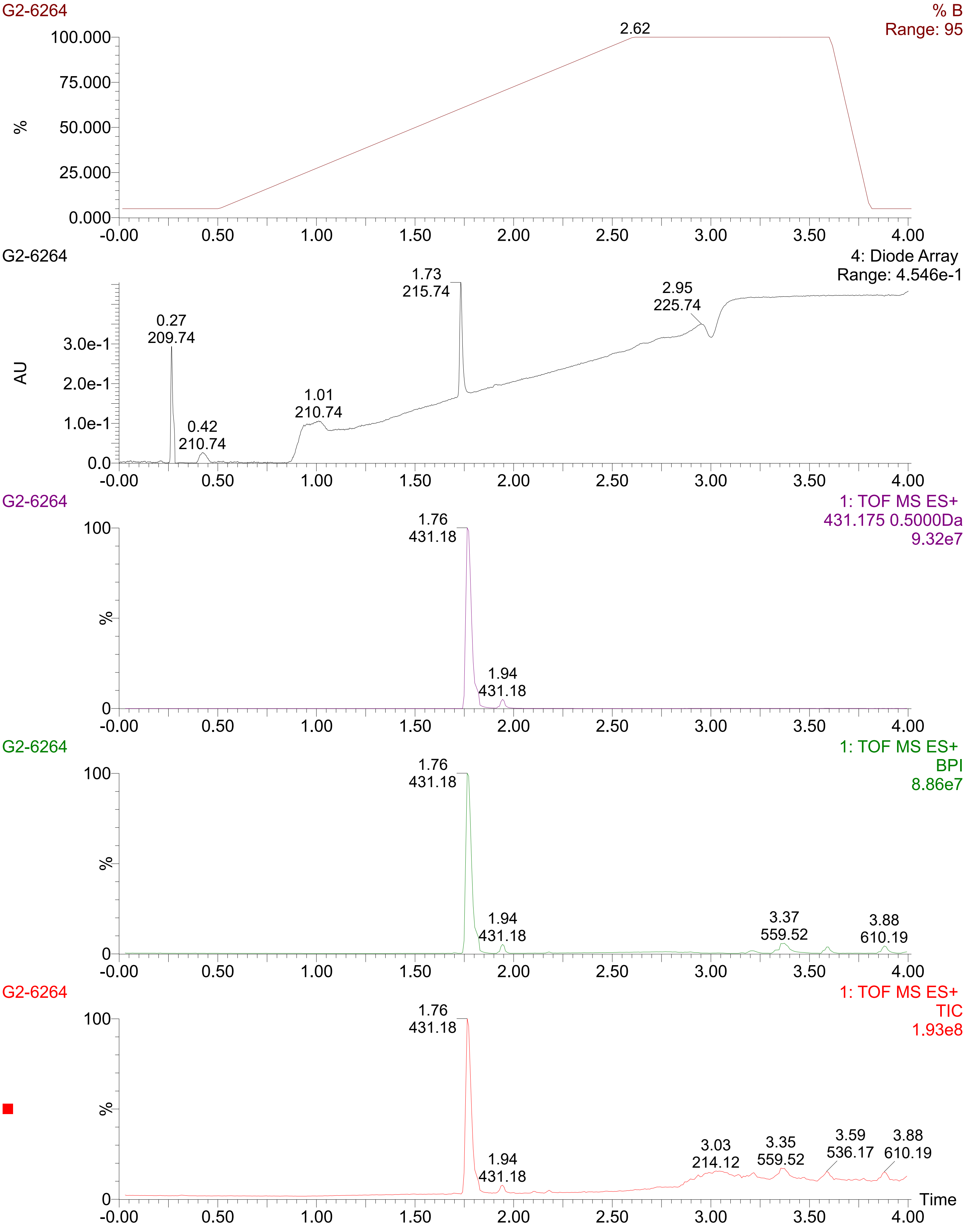

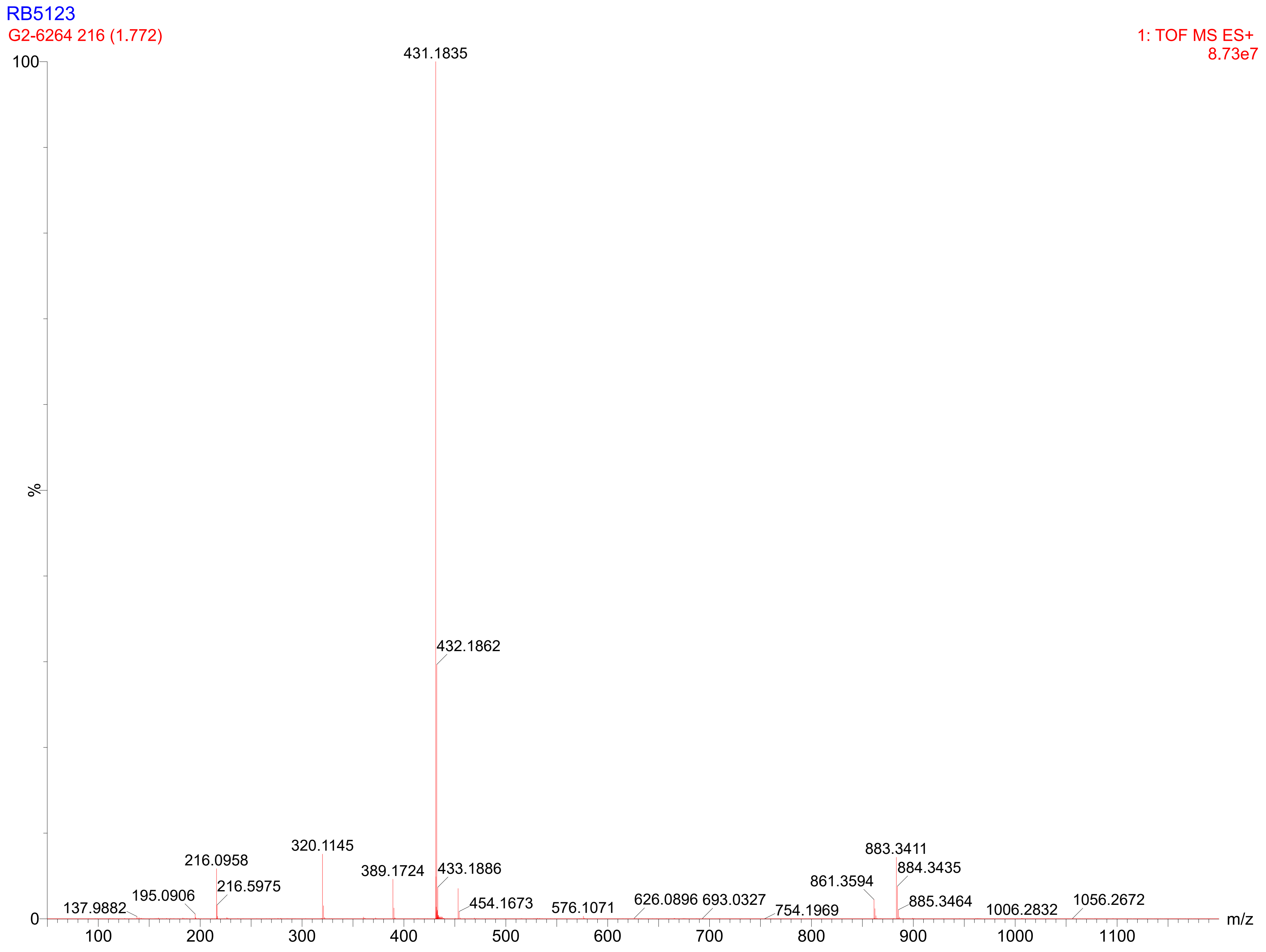

RB5123
G2-6264 216 (1.772)
1: TOF MS ES+
8.73e7
431.1835
432.1862
320.1145
216.0958
389.1724
433.1886
883.3411
884.3435
861.3594
216.5975
195.0906
137.9882
454.1673
576.1071
626.0896
693.0327
754.1969
885.3464
1006.2832
1056.2672
100
%
0
100
200
300
400
500
600
700
800
900
1000
1100
m/z

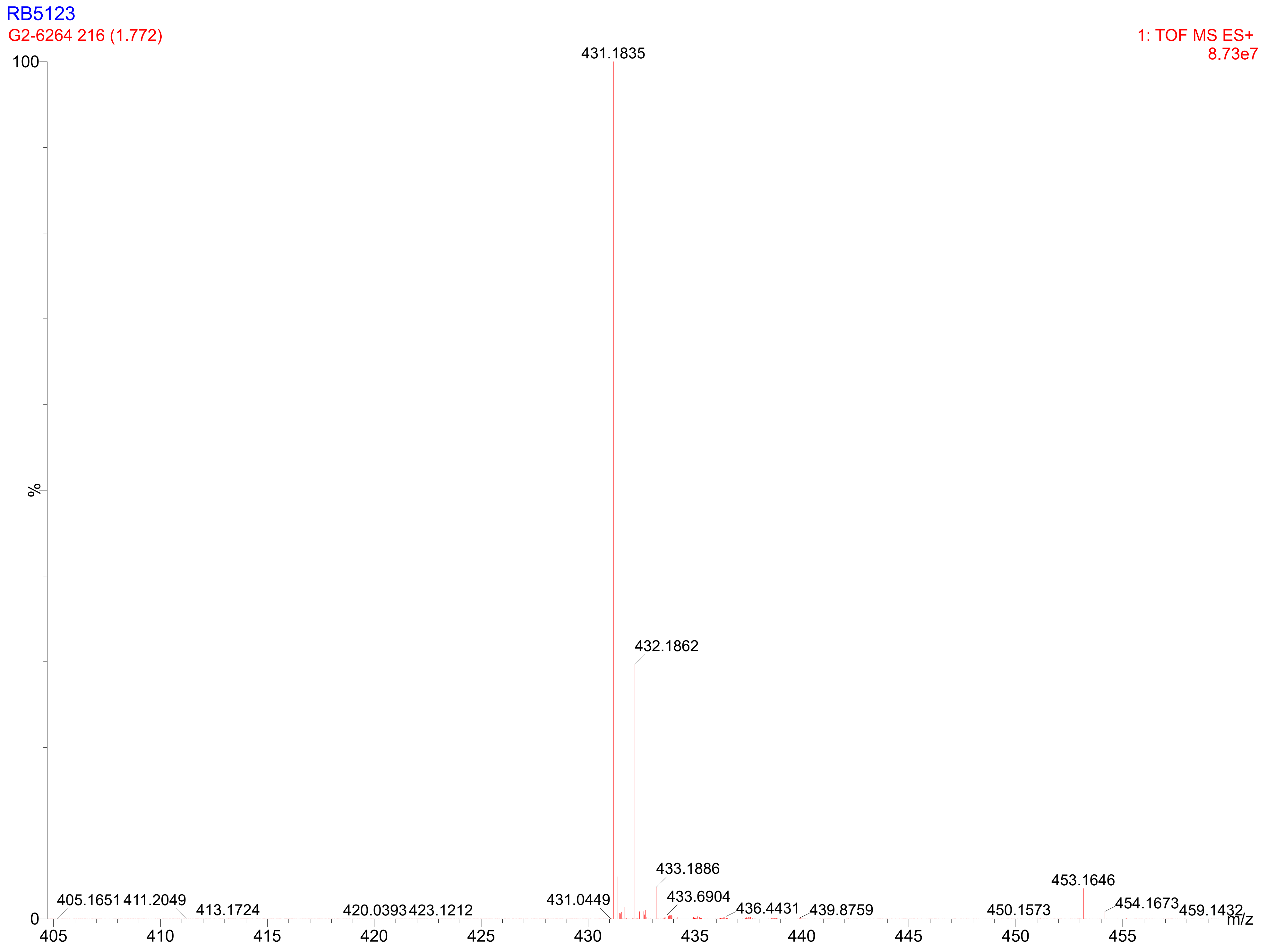

RB5123
G2-6264 216 (1.772)
1: TOF MS ES+
8.73e7
100
%
0
431.1835
432.1862
433.1886
433.6904
436.4431
439.8759
431.0449
405.1651
411.2049
413.1724
420.0393
423.1212
450.1573
453.1646
454.1673
459.1432
405
410
415
420
425
430
435
440
445
450
455
m/z

Elemental Composition Report [MH]$^{+}$

Single Mass Analysis
Tolerance = 5.0 PPM / DBE: min = -1.5, max = 100.0
Element prediction: Off
Number of isotope peaks used for i-FIT = 3

Monoisotopic Mass, Even Electron Ions
440 formula(e) evaluated with 1 results within limits (all results (up to 1000) for each mass)
Elements Used:
C: 23-23 H: 0-150 N: 0-30 O: 0-30

Minimum:				-1.5				
Maximum:		5.0	5.0	100.0				
Mass	Calc. Mass	mDa	PPM	DBE	i-FIT	Norm	Conf(%)	Formula
431.1835	431.1832	0.3	0.7	15.5	977.5	n/a	n/a	C23 H23 N6 O3

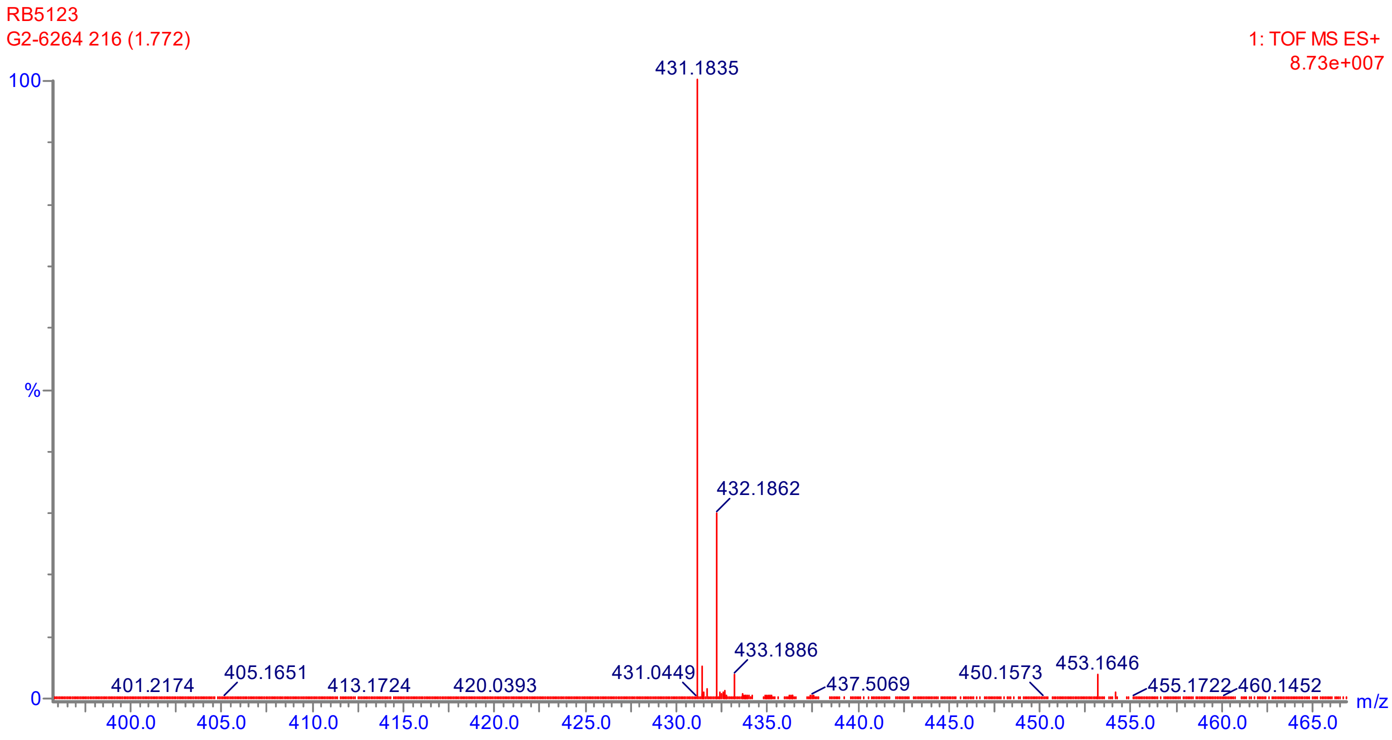

UNIVERSITY OF LEICESTER

MASS SPECTROMETRY REQUEST FORM

DEPARTMENT OF CHEMISTRY

An Open-Access mass spectrum MUST be attached for each sample submitted otherwise samples will not be run.

Name: Rob Britton **Date:** 18/06/2024

Department/section: Chemistry **Tel:** click to enter here

Supervisor: Click to enter here **UoL email:** rgb6@le.ac.uk

Sample Name:	Solvent Used:	UV Wave length for LCMS
RB5124C24H22N6O3	MeOH	215

Full Molecular Structure (insert image below)

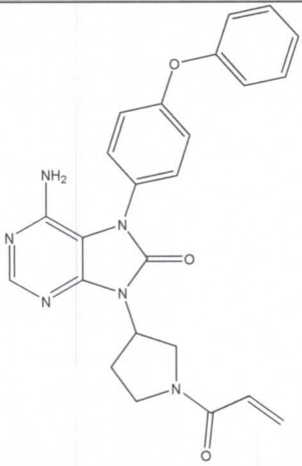

additional sample text (optional)

Molecular Formula (eg Cxx Hyy Nz *etc.*)	Molecular Weight
C24H22N6O3	442.18

Any specific sample information *(optional)*

click to enter here

Safety & handling Information: Is your compound Toxic, Hydroscopic *etc.*?

click to enter here

Analysis Request - TOF Accurate Mass

**ESI/LCMS:**	☒	**MS/MS:**	☐ Please discuss
**ASAP:** Atmopheric Solids Analvsis Probe	☐	**Custom Exp:**	☐ Please discuss
**APCI/LCMS:**	☐	**Simulation:**	☐

Operator Use Only

Date Run	Data Stored as:	Technique	Results
/ /	G #		
/ /	G #		
/ /	G #		
/ /	G #		

Comments:

***Please contact Sharad Mistry (scm11@) if you require any further help or advice**

Openlynx Report - Research-

ID:Research-2292-2 Description:RB5124_col Date:18-Jun-2024
Time:09:29:42 Method:D:\OA Methods\3_Scan_150_1000_Pos-Neg.olp
Vial:4:15 UserName:Research-

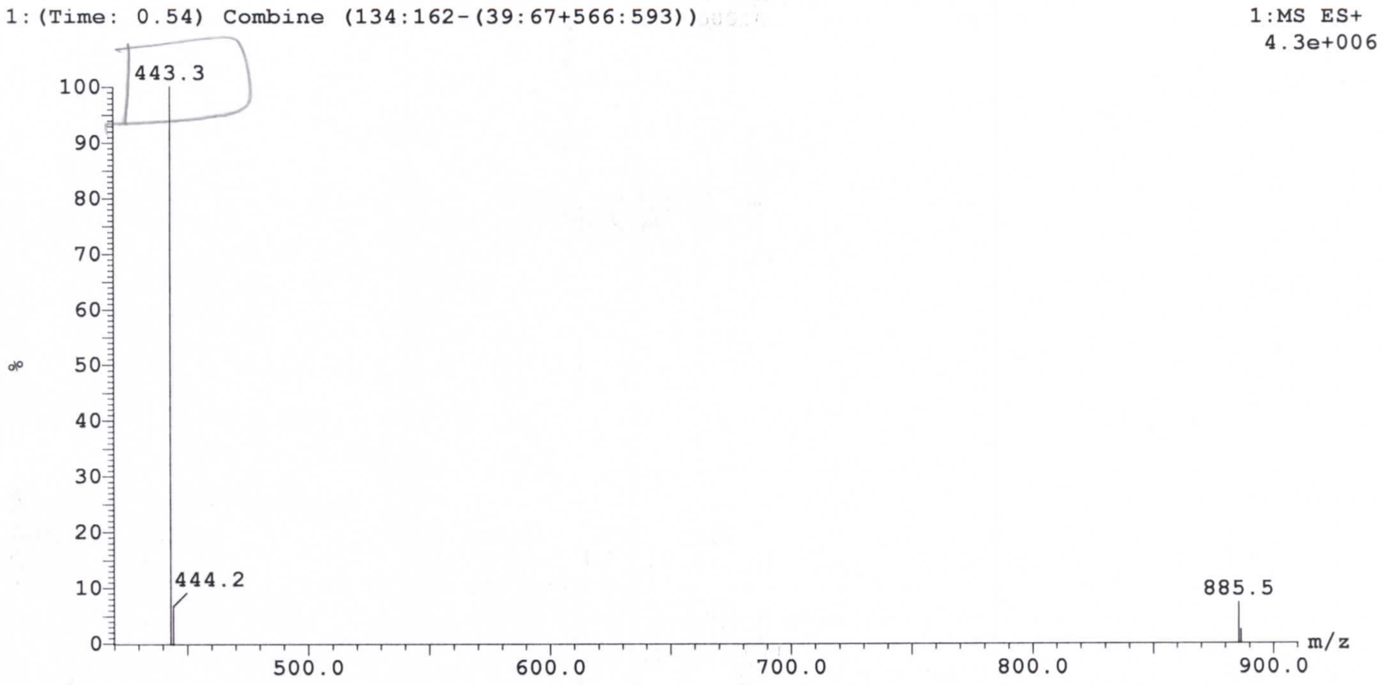

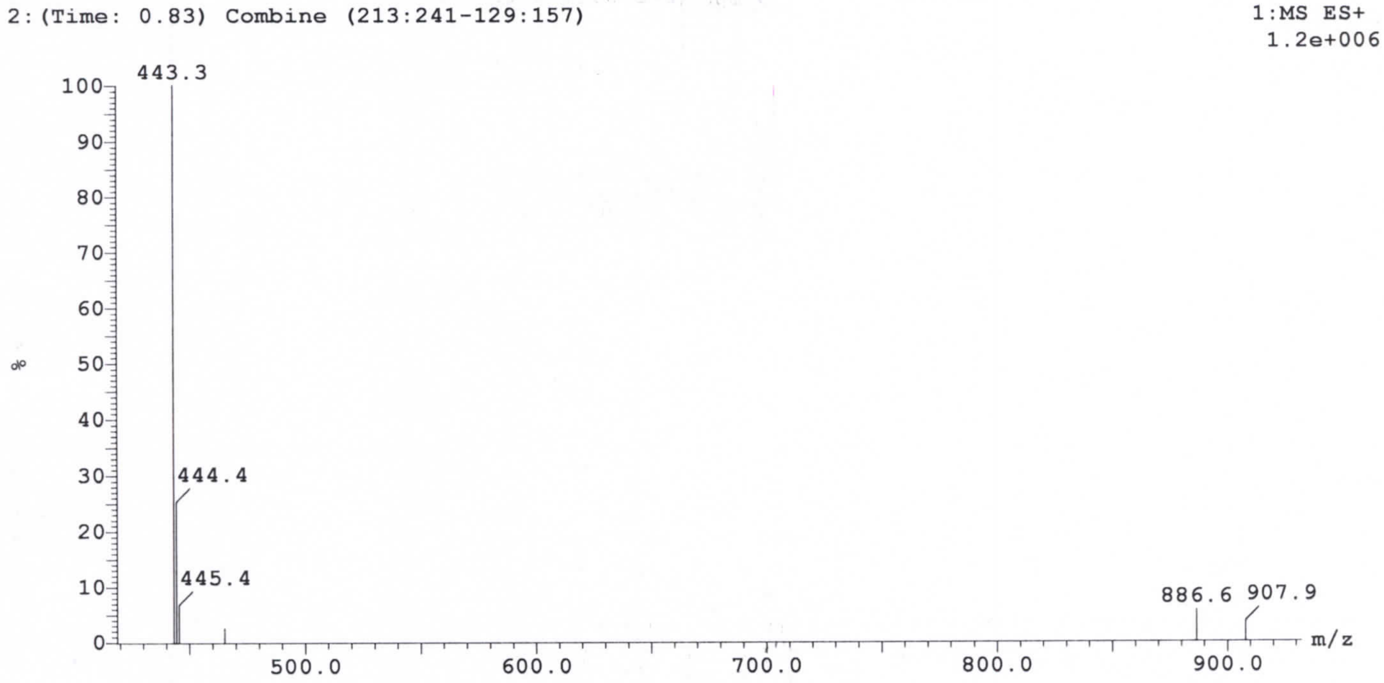

RB124

18-Jun-2024
13:54:48

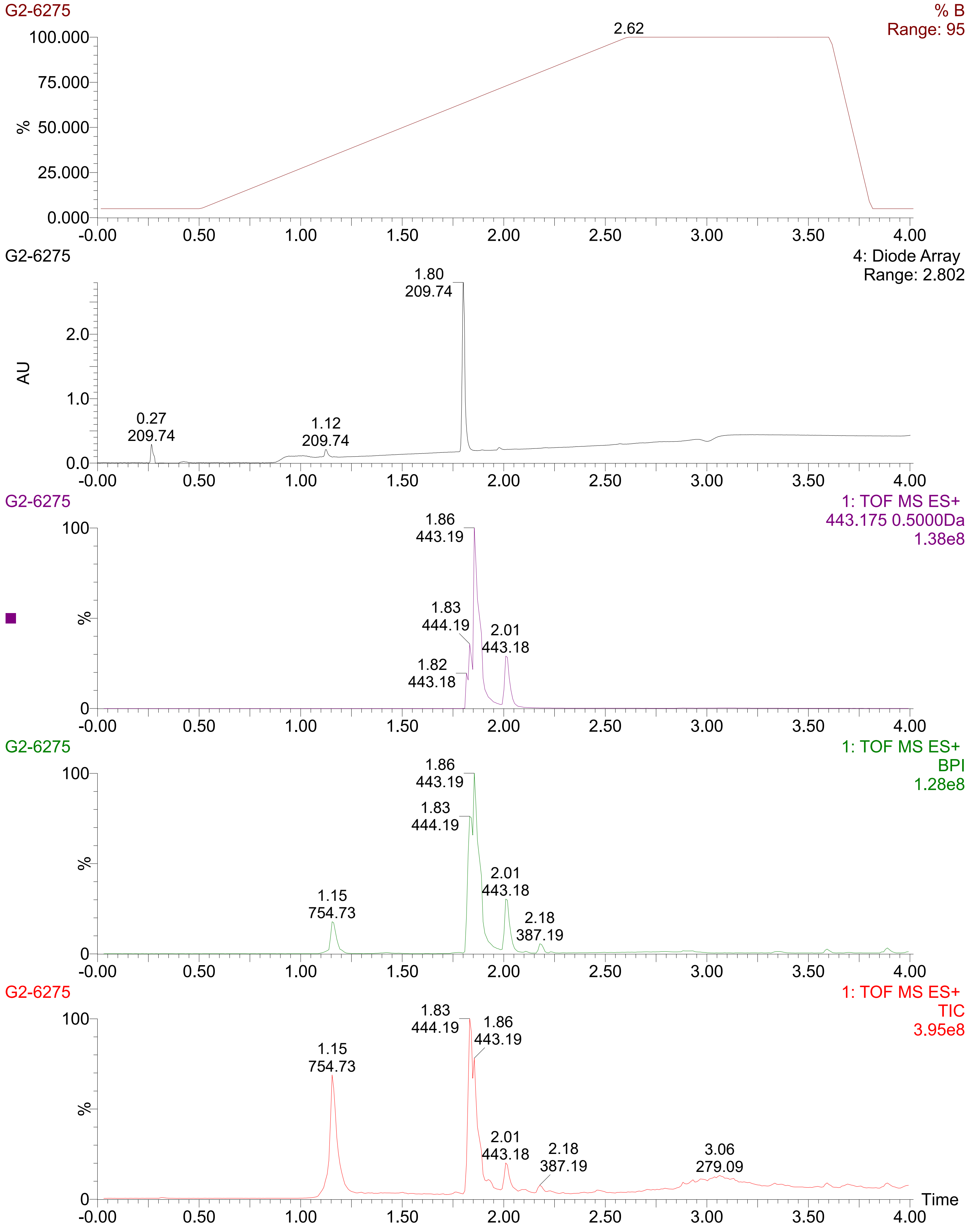

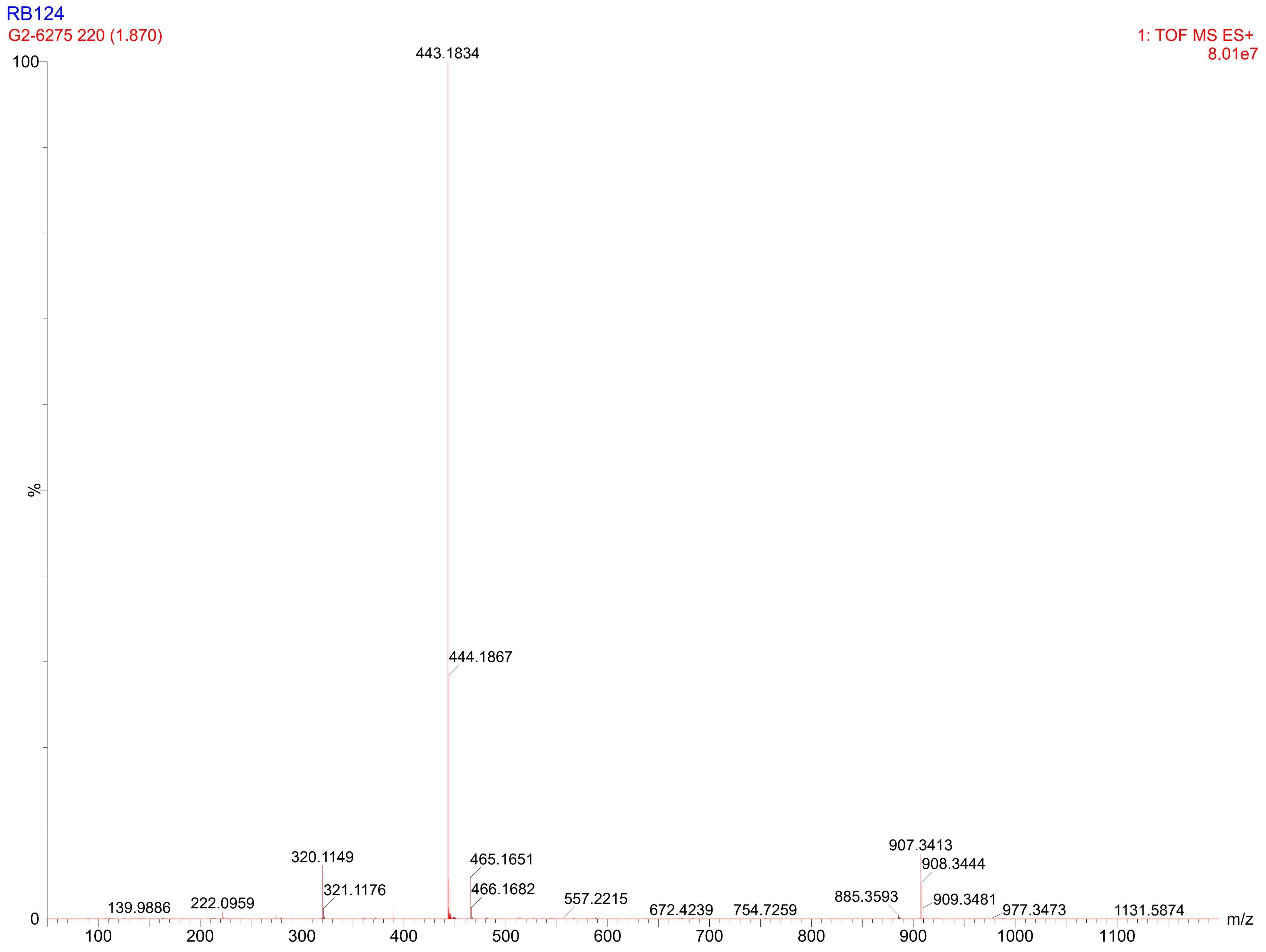
RB124
G2-6275 220 (1.870)
1: TOF MS ES+
8.01e7
443.1834
444.1867
320.1149
321.1176
465.1651
466.1682
139.9886
222.0959
557.2215
672.4239
754.7259
885.3593
907.3413
908.3444
909.3481
977.3473
1131.5874
100
%
0
100
200
300
400
500
600
700
800
900
1000
1100
m/z

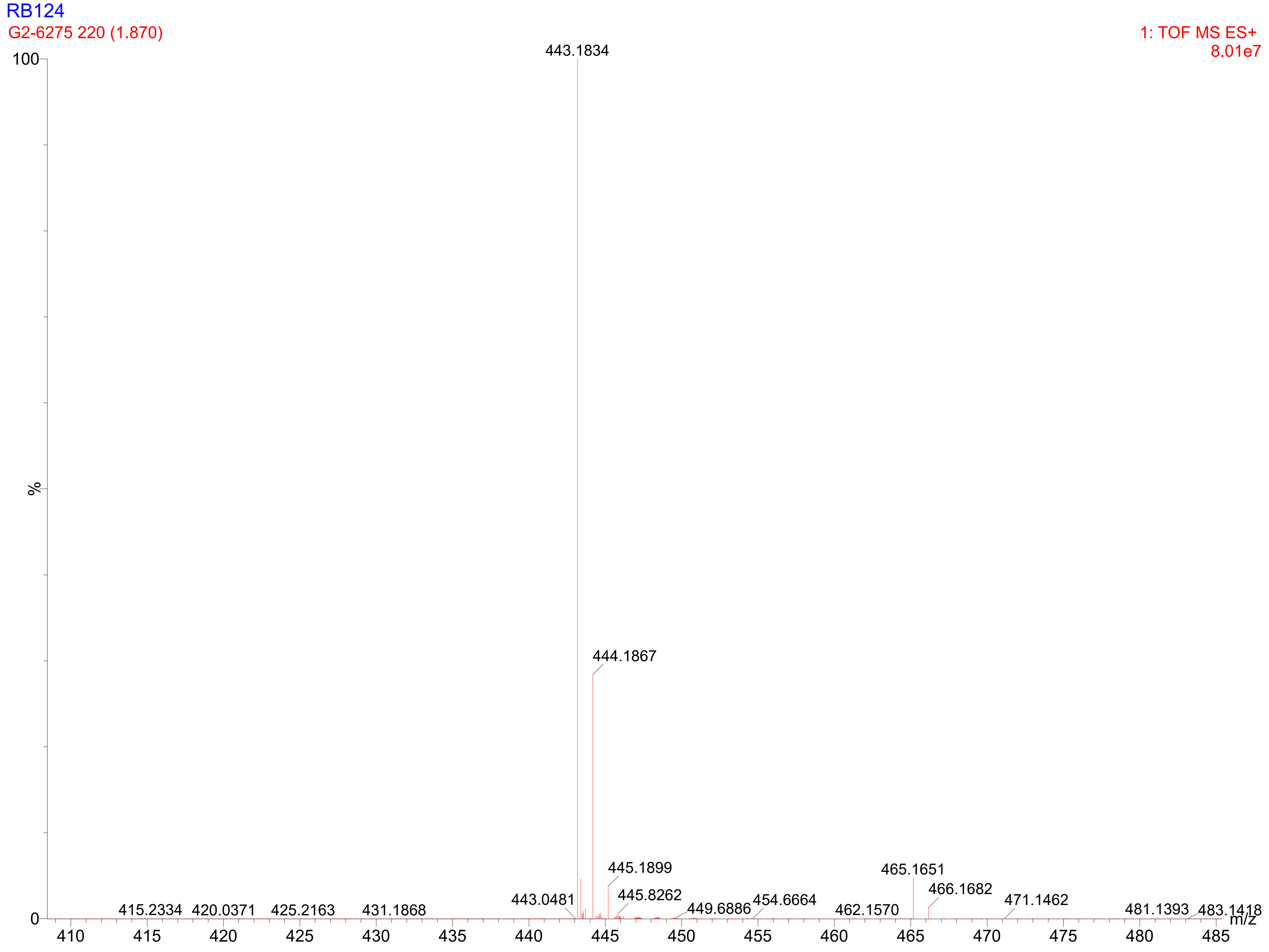

RB124
G2-6275 220 (1.870)
1: TOF MS ES+
8.01e7
443.1834
444.1867
445.1899
445.8262
443.0481
449.6886
454.6664
465.1651
466.1682
471.1462
415.2334
420.0371
425.2163
431.1868
462.1570
481.1393
483.1418
100
%
0
m/z
410
415
420
425
430
435
440
445
450
455
460
465
470
475
480
485

Elemental Composition Report [MH]$^{+}$

Single Mass Analysis
Tolerance = 5.0 PPM / DBE: min = -1.5, max = 100.0
Element prediction: Off
Number of isotope peaks used for i-FIT = 3

Monoisotopic Mass, Even Electron Ions
463 formula(e) evaluated with 1 results within limits (all results (up to 1000) for each mass)
Elements Used:
C: 24-24 H: 0-150 N: 0-30 O: 0-30

Minimum:				-1.5				
Maximum:		5.0	5.0	100.0				
Mass	Calc. Mass	mDa	PPM	DBE	i-FIT	Norm	Conf(%)	Formula
443.1834	443.1832	0.2	0.5	16.5	981.3	n/a	n/a	C24 H23 N6 O3

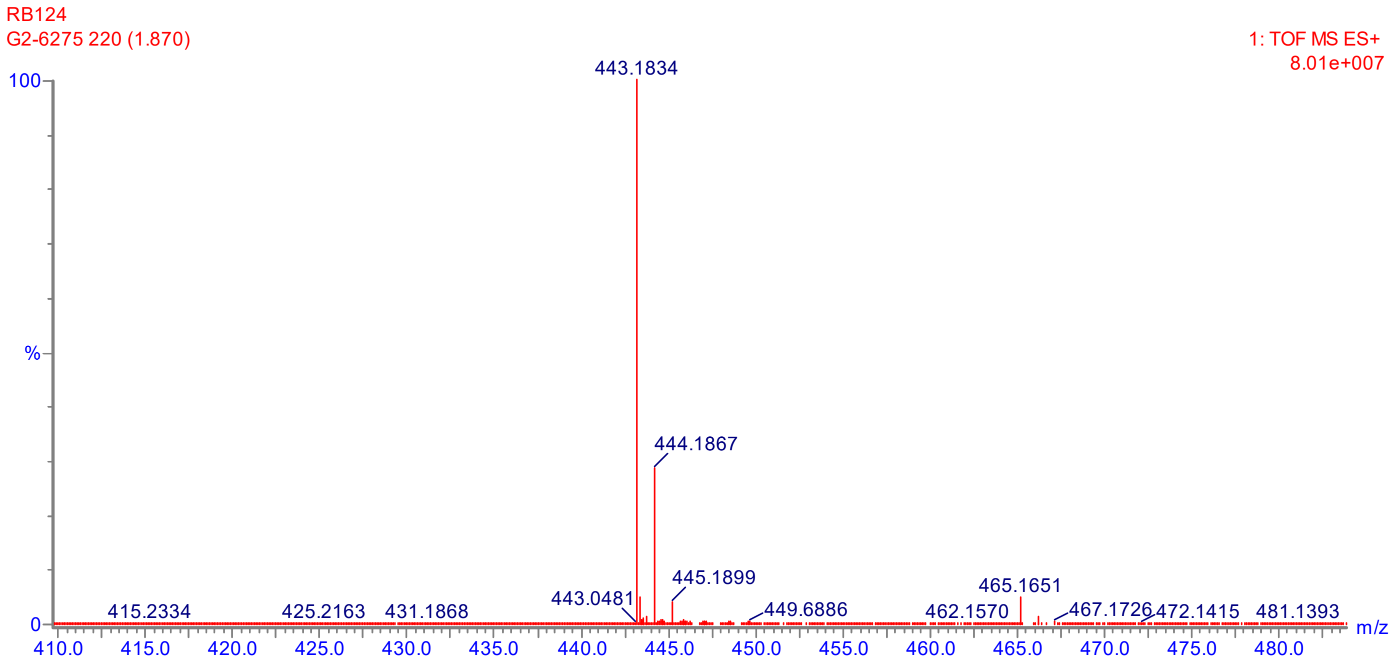

Supplement: Supplement 2 — Supp. Data File 2: PDF file containing supporting NMR and MS data for acrylamide-Tirabrutinib, 2-butynamide-Ibrutinib and ‘alkane’ analog synthesis. [file media-2.pdf]
